# Neonatal microbiota colonization primes maturation of goblet cell–mediated protection in the pre-weaning colon

**DOI:** 10.1084/jem.20241591

**Published:** 2025-05-05

**Authors:** Åsa Johansson, Mahadevan Venkita Subramani, Bahtiyar Yilmaz, Elisabeth E.L. Nyström, Elena Layunta, Liisa Arike, Felix Sommer, Philip Rosenstiel, Lars Vereecke, Louise Mannerås-Holm, Andy Wullaert, Thaher Pelaseyed, Malin E.V. Johansson, George M.H. Birchenough

**Affiliations:** 1 Department of Medical Biochemistry and Cell Biology, Institute of Biomedicine, University of Gothenburg, Gothenburg, Sweden; 2 Wallenberg Centre for Molecular and Translational Medicine, University of Gothenburg, Gothenburg, Sweden; 3 Department for BioMedical Research, University of Bern, Bern, Switzerland; 4 Institute of Clinical & Molecular Biology, University of Kiel, Kiel, Germany; 5 VIB-UGent Center for Inflammation Research, Ghent, Belgium; 6 Department of Internal Medicine and Pediatrics, Ghent University, Ghent, Belgium; 7 Wallenberg Laboratory, Institute of Medicine, University of Gothenburg, Gothenburg, Sweden; 8 Department of Biomedical Sciences, Cell Death Signalling Lab, University of Antwerp, Antwerp, Belgium

## Abstract

Regulated host–microbe interactions are a critical aspect of lifelong health. Colonic goblet cells protect from microorganisms via the generation of a mucus barrier structure. Bacteria-sensing sentinel goblet cells provide secondary protection by orchestrating mucus secretion when microbes breach the mucus barrier. Mucus deficiencies in germ-free mice implicate a role for the microbiota in programming barrier generation, but its natural ontogeny remains undefined. We now investigate the mucus barrier and sentinel goblet cell development in relation to postnatal colonization. Combined in vivo and ex vivo analyses demonstrate rapid and sequential microbiota-dependent development of these primary and secondary goblet cell protective functions, with dynamic changes in mucus processing dependent on innate immune signaling via MyD88 and development of functional sentinel goblet cells dependent on the NADPH/dual oxidase family member Duox2. Our findings identify new mechanisms of microbiota–goblet cell regulatory interaction and highlight the critical importance of the pre-weaning period for the normal development of protective systems that are key legislators of host–microbiota interaction.

## Teaser

The colonic mucus barrier and sentinel goblet cells protect the distal gut from microbial challenges. This study demonstrates that microbial colonization drives sequential development of both of these barrier mechanisms in the pre-weaning intestine, setting the stage for the increased microbial burden associated with weaning.

## Graphical Abstract

**Figure:**
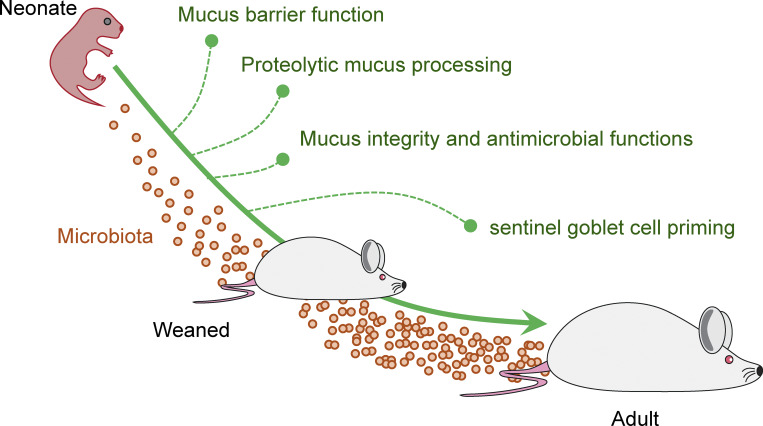

## Introduction

A neonatal “window of opportunity” where phased interactions between the newborn and colonizing microorganisms occur is essential to establish lifelong immunological homeostasis (Torow and Hornef, 2017). Intestinal mucosal immune responses to the increased bacterial load and diversity associated with weaning (the “weaning reaction”) are associated with healthy imprinting that protects against inflammatory challenges in later life (Al Nabhani et al., 2019). While the hematopoietic aspects of this development are the subject of intensive study, the development of mucosal epithelial barrier systems that serve as the proximal interface with colonizing microorganisms remains less well-defined. A key innate defensive system that protects the intestinal mucosal epithelium is the dynamic mucus barrier structure secreted by epithelial goblet cells (GCs) (Gustafsson and Johansson, 2022). Crucially, the developmental ontogeny of this barrier system and other GC-intrinsic defensive functions remain almost completely unknown.

The mucus barrier is structurally defined by the polymeric gel-forming Mucin-2 (Muc2) glycoprotein, and its genomic deletion results in increased susceptibility to infection (Bergstrom et al., 2010), inflammation (Van der Sluis et al., 2006), and tumorigenesis (Velcich et al., 2002), underlining the crucial role of mucus as a legislator of intestinal homeostasis. The colonic mucus system has evolved to physically contain and segregate the intestinal microbiota by forming a dense, tissue-adherent inner mucus layer (IML) barrier that is impenetrable to most microbes (Johansson et al., 2008). The Muc2 polymers that form the IML are protected from microbial degradation by heavy O-glycosylation (Bergstrom et al., 2017), intermolecular mucin isopeptide cross-linking (Sharpen et al., 2022), and the presence of proteins that aggregate or kill mucus-invasive bacteria (Bergström et al., 2016; Bülck et al., 2023; Propheter et al., 2017). IML Muc2 polymers undergo postsecretory processing by endogenous proteases (Nyström et al., 2018) that expand the polymeric network and likely contribute toward fecal encapsulation of the colonic microbiota (Bergstrom et al., 2020). Consequently, the IML is the primary GC-intrinsic protective system in the colon, and its protective functions are strongly influenced by several layers of posttranslational Muc2 modification.

Besides IML formation, colonic GCs have additional proposed roles in mucosal protection. A bacteria-sensing subpopulation of upper crypt GCs referred to as sentinel GCs (senGCs) orchestrates defensive mucus secretion in response to elevated levels of specific TLR ligands via assembly of an NLR family pyrin domain containing 6 (Nlrp6)–dependent inflammasome complex (Birchenough et al., 2016). This process is believed to function as a secondary protective function that guards colonic crypts against bacteria that have penetrated the IML. Furthermore, GCs can act as GC-associated antigen passages to transfer luminal material to lamina propria immune cells and thereby regulate mucosal immunity against dietary and microbial antigens (Gustafsson et al., 2021; Kulkarni et al., 2020), thus illustrating that GCs have protective functions beyond constitutive mucus secretion.

While knowledge of GC protective functions continues to expand, our understanding of how and when these systems are established during early life development is incomplete. Postnatal mucus barrier development in the small intestine varies along the proximal-distal axis, but the regulatory mechanisms governing this heterogeneity are unknown (Birchenough et al., 2017). Prior work indicates that colonic GC-associated antigen passages are induced during the pre-weaning phase and repressed during the suckling-weaning transition (Knoop et al., 2017); however, postnatal IML or senGC functional maturation has not been described. Studies in adult germ-free (GF) mice indicate that IML functions are compromised in the absence of a microbiota (Bergstrom et al., 2020; Jakobsson et al., 2015; Johansson et al., 2015), thus hinting that these protective functions are likely to develop during establishment of the colonic microbiota in the neonatal-weaning period. We have therefore sought to define the postnatal developmental dynamics of colonic IML and senGC maturation and examine their relation to microbiota colonization to define the maturation of GC-intrinsic protective systems in the large intestine and thereby establish how these systems contribute to host-microbiota homeostasis during the neonatal window of opportunity.

## Results

### The colonic IML matures in the pre-weaning environment

**Figure 1.**
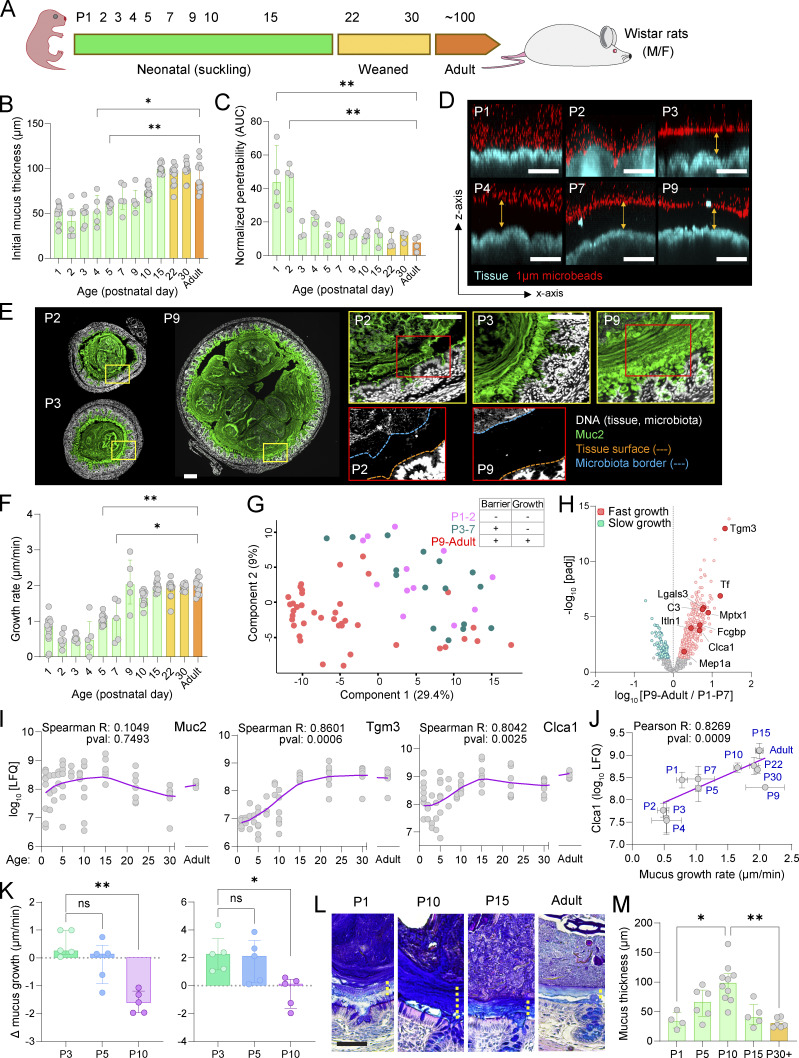
**Postnatal maturation of the colonic IML barrier. (A)** Sampling time points in postnatal days (P) of neonatal, weaned, and adult Wistar rats. **(B)** IML thickness quantified by ex vivo needle measurement. **(C)** IML barrier function quantified by ex vivo microbead penetration. **(D)** Representative confocal z-stacks used to generate data shown in C. Images show x/z-axis cross-sections of colonic tissues overlaid with microbeads. Impenetrable mucus is indicated (yellow arrows). **(E)** Confocal micrographs of fixed colonic tissue sections stained for DNA (grey) and Muc2 (green). Dashed lines in high-magnification P2 and P9 images show tissue surface (orange) and microbiota border (blue). **(F)** IML growth rate quantified by ex vivo needle measurement. **(G)** Principal component analysis plot of complete mucus proteome data. Samples are color coded based on age-dependent IML ex vivo phenotype: P1–P2 (low barrier function, slow growth; pink), P3–P7 (high barrier function, slow growth; teal), and P9–Adult (high barrier function, fast growth; red). **(H)** Volcano plot comparing complete mucus proteome data from slow growth (P1–P7) and fast growth (P9–Adult) samples. Proteins significantly enriched in slow (teal) or fast (red) growth groups are indicated. **(I)** Abundance of Muc2 (left), Tgm3 (middle), and Clca1 (right) proteins at different ages from label-free quantification (LFQ) of complete mucus proteome data. LOWESS curves (purple) are indicated for each dataset. Spearman correlation R and P values derived from correlating age and protein LFQ are shown. **(J)** Correlation of ex vivo mucus growth rate and Clca1 mucus proteome abundance (LFQ) at different ages. Simple linear regression (purple) is indicated. Pearson correlation R and P value derived from correlating mucus growth rate and Clca1 LFQ are shown. **(K)** Ex vivo mucus growth and response to metalloprotease inhibition by EDTA (left) or Comp PIC (right) treatment at different ages. Graphs show changes in mucus growth rate (Δ mucus growth; right) in response to inhibitor treatment. **(L)** AB/PAS-stained distal colon tissue sections from animals at different ages. Yellow dashed line indicates the IML. **(M)** Quantification of IML thickness values at different ages based on images shown in L. Data represent *n* = 5–19 (A–J, L, and M) or *n* = 5 (K) animals per group, as indicated. All data are pooled from at least three independent litters. All error-bar graphs show median and interquartile range. Statistical comparisons between groups by Kruskal–Wallis and Dunn’s multiple comparison (B, C, F, K, and M) or Welch’s *t* test and Benjamini–Hochberg FDR correction (H); P < 0.05 (*), <0.01 (**). Image scale bars are 100 µm. AUC, area under the curve.

The colonic IML is the primary GC-intrinsic protective system (Johansson et al., 2008). The IML comprises a tissue-adherent layer processed in part by the action of endogenous metalloproteases (Nyström et al., 2018) and stool-associated layer that encapsulates the fecal microbiota (Bergstrom et al., 2020). These elements can be quantified either by ex vivo or in vivo (histology) approaches, respectively. Postnatal IML development was analyzed in Wistar rats across neonatal (postnatal day [P]1–P15), weaned (P22–P30), and adult (>P100) stages (Fig. 1 A). Rats were employed to provide sufficient tissue for ex vivo analysis that would not be feasible with neonatal mice. A tissue-adherent mucus layer was present at all ages, with thickness increasing from ∼50 μm at P1 to ∼100 μm at P15, maintained into adulthood (Fig. 1 B). However, barrier function transitioned abruptly from a penetrable IML at P1–2 to an impenetrable, adult-like structure at P3 (Fig. 1, C and D). Histological immunostaining for Muc2 confirmed IML formation, revealing a stratified, stool-associated structure encapsulating the microbiota from P3 onward, absent at P2 (Fig. 1 E). Despite the underdeveloped IML at P2, no microbiota–epithelial contact was observed. Postsecretion IML processing was assessed via ex vivo mucus growth rate, showing fluctuations in the neonatal period: ∼1 µm/min at P1, dropping to ∼0.5 µm/min at P2–P4, then rising to ∼2 µm/min between P5–P10 and maintained into adulthood (Fig. 1 F). Combined, these findings indicate that IML formation occurs primarily pre-weaning, with dynamic changes in thickness, barrier function, and processing in early postnatal life.

### Postnatal IML maturation reflects mucus compositional and processing dynamics

To identify molecular regulators of IML maturation, we performed label-free mass spectrometry of the IML proteome, revealing age-dependent variability in mucus protein composition. Principal component analysis and grouping samples into three phenotypes: P1–2 (penetrable, low growth), P3–P7 (impenetrable, low growth), and P9–adult (impenetrable, high growth) identified clustering by growth rate. (Fig. 1 G). Comparing high-growth (P9–adult) and low-growth (P1–7) samples identified enrichment of antimicrobial proteins (e.g., Itln1, Mptx1, Lgals3, Tf, and C3) (Fig. 1 H). Muc2 abundance followed a bell-shaped pattern, peaking at P15 and declining by P30. In contrast, two key Muc2-processing enzymes transglutaminase 3 (Tgm3) that regulates IML integrity (Sharpen et al., 2022) and the metalloprotease calcium-activated chloride channel regulator 1 (Clca1) (Nyström et al., 2018) both significantly and positively correlated with age (Fig. 1 I).

**Figure S1.**
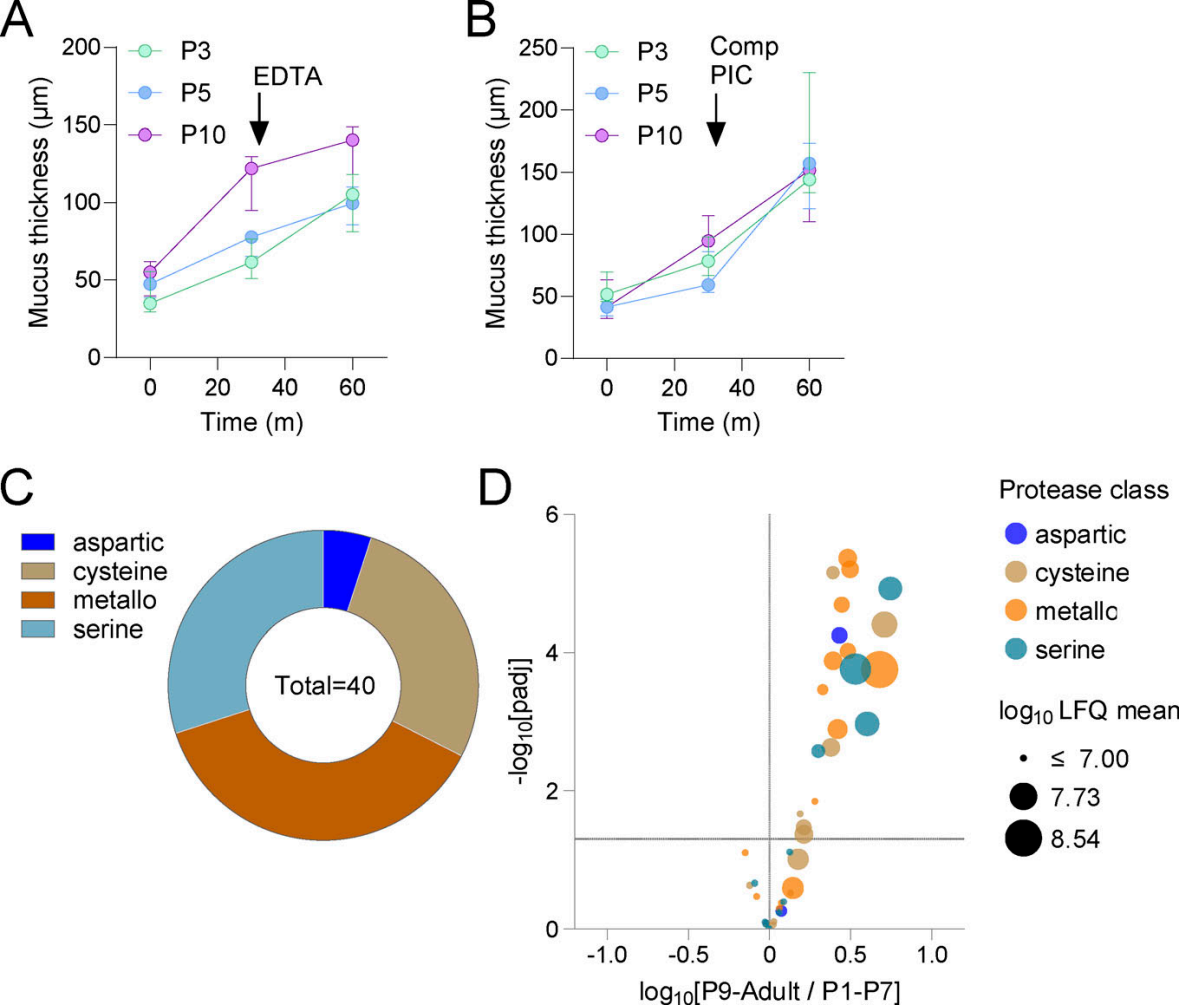
**R** **elated to** Fig. 1 **. (A)** Ex vivo mucus growth and response to metalloprotease inhibition by EDTA treatment at different ages. Graph show mucus thickness values over time in response to EDTA treatment at *t* = 30 min (m). **(B)** Ex vivo mucus growth and response to serine/cysteine protease inhibition by Comp PIC treatment at different ages. Graph shows mucus thickness values over time in response to Comp PIC at *t* = 30 m. **(C)** Proteases belonging to different classes detected in mucus proteome data of at least one age group. **(D)** Volcano plot comparing protease abundance in slow growth (P1–P7) and fast growth (P9–Adult) samples. Proteases color coded by class; data points size proportional to label-free quantification (LFQ) abundance values. Data represent *n* = 5 (A and B) or *n* = 3–16 (C and D) animals per group. All error-bar graphs show median and interquartile range. All data are pooled from at least two independent litters or experiments.

Clca1 drives proteolytic mucus expansion and ex vivo mucus growth (Nyström et al., 2018), likely playing a role in releasing Muc2 polymers to coat and encapsulate the fecal microbiota (Bergstrom et al., 2020). Clca1 mucus abundance positively correlated with mucus growth rates (Fig. 1 J), prompting us to investigate its role using EDTA, a known Clca1 inhibitor (Nyström et al., 2018). While EDTA had no effect on low-growth neonatal samples (P3/5), it significantly inhibited mucus growth in high-growth (P10) samples, indicating Clca1’s role in postnatal mucus processing (Fig. 1 K and Fig. S1 A). Unexpectedly, pan-serine/cysteine protease inhibition (complete protease inhibitor cocktail [Comp PIC]) increased mucus growth in P3/5 tissues, suggesting active suppression of mucus expansion in early neonates, whereas Comp PIC had no impact on Clca1-dependent P10 mucus growth, as previously reported for adults (Nyström et al., 2018) (Fig. 1 K and Fig. S1 B). Mucus proteomics identified 40 proteases of all classes in at least one age group (Fig. S1 C); however, none were enriched in mucus from younger tissues (Fig. S1 D), preventing us from identifying the protease(s) suppressing mucus growth based purely on abundance. We hypothesized that proteolytic inhibition of mucus expansion may initially thicken the stool-associated IML, which later transitions to Clca1-driven mucus release. Supporting this, alcian blue/periodic acid-Schiff (AB/PAS) staining showed significant IML thickening between P1–P10, which decreased after P15 toward the adult state. (Fig. 1, L and M).

These experiments suggest that postnatal IML maturation is driven by increased levels of Muc2-processing proteins (e.g., Clca1), alongside factors reinforcing barrier integrity (Tgm3) and antimicrobial defense. Notably, low mucus growth in early neonates appears to result from serine/cysteine protease activity, revealing unexpected active suppression of mucus expansion. The concurrent thickening of the stool-associated IML in vivo suggests that dynamic proteolytic processing plays a key role in establishing a robust IML barrier in early postnatal development.

### Microbiota colonization and MyD88 signaling regulate specific aspects of IML maturation

**Figure 2.**
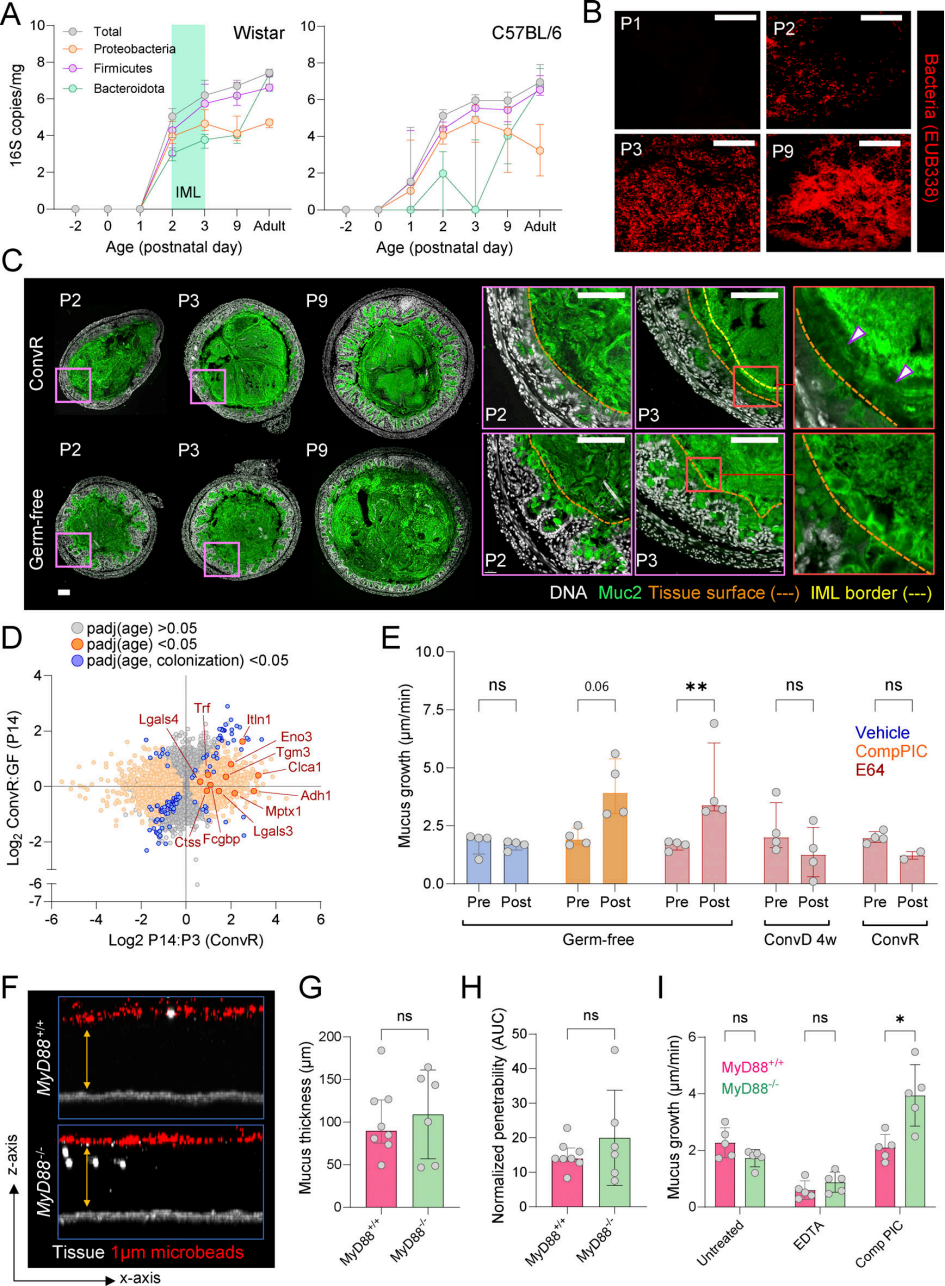
**Postnatal IML maturation is driven by both microbiota-dependent and -independent factors. (A)** Total and taxon-specific colonic bacterial load in fetal and postnatal Wistar rats (left) and C57BL/6 mice (right) quantified by 16S qPCR of stool DNA. IML formation period between P2–P3 in rats is indicated. **(B)** Confocal micrographs of fixed mouse colonic tissue stained for luminal bacteria by 16S FISH. **(C)** Confocal micrographs of fixed colonic tissue sections stained for DNA (grey) and Muc2 (green). Dashed lines in high-magnification images (purple boxes) show tissue surface (orange) and IML border (yellow). Stratified Muc2 layers present in ConvR but not GF P3 IML are indicated (arrowheads) in highest magnification images (red boxes). **(D)** Comparison of gene expression ratios between ConvR P14:P3 mice (age dependent) and P14 ConvR:GF mice (microbiota dependent) quantified by DESeq2 analysis of bulk colonic RNA sequencing data. Genes significantly regulated by age (orange) or by both age and colonization status (blue) are indicated. **(E)** Ex vivo mucus growth in GF, ConvD, or ConvR tissue before (pre) and after (post) serine/cysteine protease inhibition by Comp PIC or the cysteine protease-specific inhibitor E64. **(F)** Ex vivo analysis of MyD88^+/+^ and MyD88^−/−^ IML thickness and barrier function by microbead penetration. Images are x/z-axis cross-sections of confocal z-stacks showing colonic tissues (grey) overlaid with microbeads (red). Impenetrable mucus is indicated (yellow arrows). **(G)** Ex vivo analysis of MyD88^+/+^ and MyD88^−/−^ IML thickness based on images shown in F. **(H)** Ex vivo analysis of MyD88^+/+^ and MyD88^−/−^ IML barrier function based on images shown in F. **(I)** Ex vivo mucus growth in MyD88^+/+^ and MyD88^−/−^ tissue in response to metalloprotease (EDTA) or serine/cysteine protease (Comp PIC) inhibition. Data represent *n* = 4–5 (A–C, E, and I), *n* = 6–8 (F–H), or *n* = 3 (D) animals per group, as indicated. All data are pooled from at least two independent litters or experiments. All error-bar graphs show median and interquartile range. Statistical comparisons between groups by DESeq2 (D), Kruskal–Wallis and uncorrected Dunn’s test (E), or Mann–Whitney test (G–I); P < 0.05 (*), <0.01 (**). Image scale bars are 50 µm (B) or 100 µm (C). AUC, area under the curve.

Previous studies have shown that IML function and composition in adult mice can be influenced by the intestinal microbiota (Arike et al., 2020; Jakobsson et al., 2015; Johansson et al., 2015); however, whether this applies to natural postnatal IML formation remains unclear. To investigate, we compared neonatal colonic tissue from conventionally raised (ConvR) and GF C57BL/6 mice over P1–P9 using an in vivo approach to complement data obtained from rats (Fig. 1). To confirm similar postnatal colonization dynamics in mice and rats, we quantified microbiota colonization via 16S quantitative PCR (qPCR), detecting total and taxon-specific bacterial load (Proteobacteria, Firmicutes, and Bacteroidetes) in prenatal and postnatal stool samples. In both species, bacterial colonization became evident at P1–P2, initially dominated by Firmicutes and Proteobacteria, with Bacteroidetes increasing subsequently (Fig. 2 A). 16S fluorescence in situ hybridization (FISH) staining of neonatal mouse luminal content confirmed that IML barrier formation (observed in rats at P2–P3) occurred immediately after initial microbiota seeding of the colon (Fig. 2 B).

**Figure S2.**
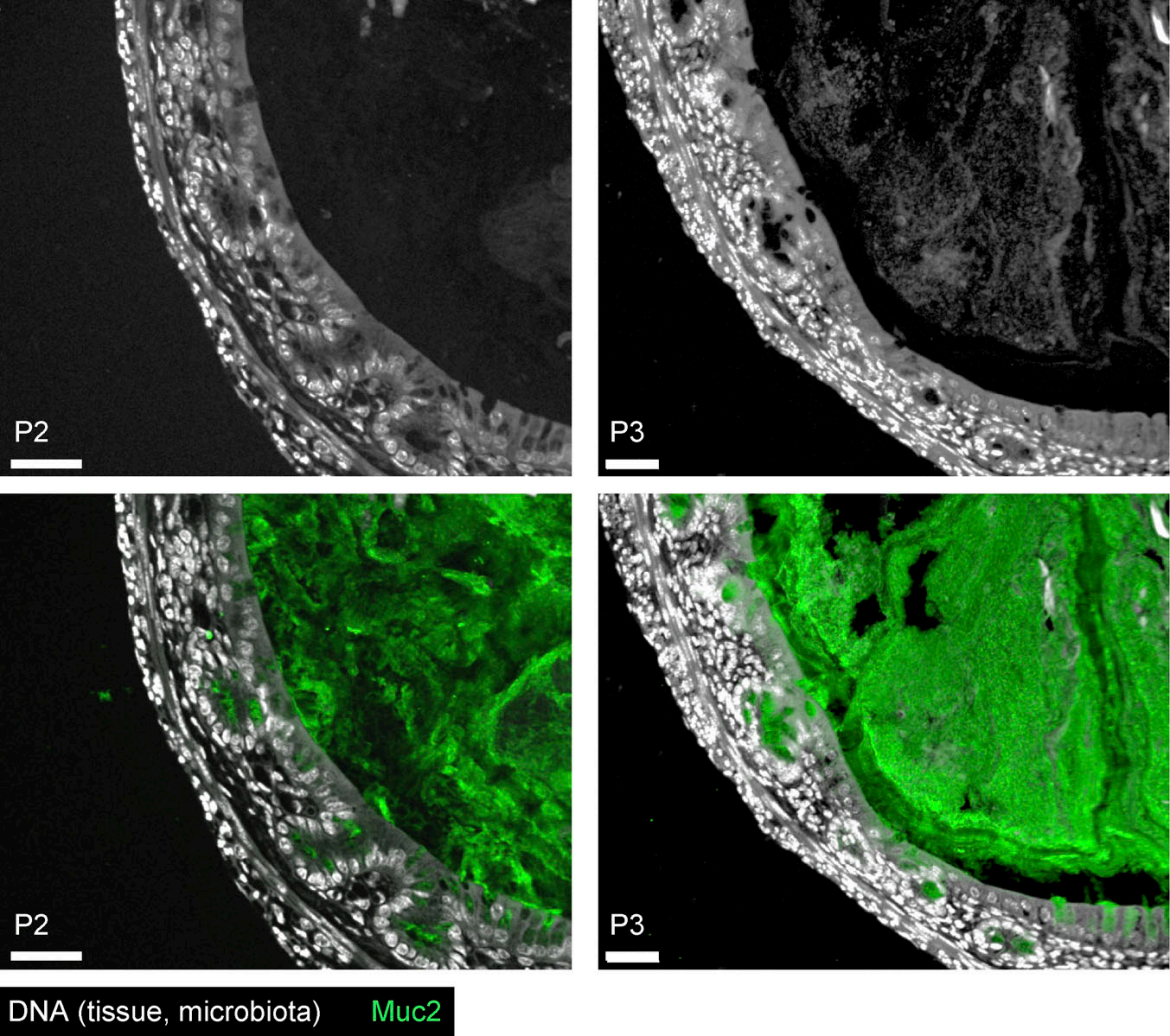
**R** **elated to** Fig. 2 **.** Confocal micrographs of fixed colonic tissue sections from ConvR P2 (left) and P3 (right) mice stained for DNA (grey) and Muc2 (green). Images are representative of *n* = 3–5 animals per group pooled from two independent litters. Image scale bars are 40 µm.

Due to tissue size limitations, ex vivo mucus analysis is not feasible in neonatal mice, so IML formation was assessed histologically via Muc2 immunostaining in ConvR and GF neonates. Both groups showed comparable luminal Muc2 levels at all ages, with no clear organization at P2. By P3, ConvR mice exhibited a stratified IML barrier encapsulating the microbiota, absent in age-matched GF mice, indicating microbiota-dependent IML development similar to that observed in rats (Fig. 2 C and Fig. S2).

Since EDTA inhibits GF mucus growth to the same extent as ConvR tissues, Clca1-dependent mucus processing appears microbiota independent (Nyström et al., 2018). To investigate this further, we performed bulk RNA sequencing on ConvR P3, ConvR P14, and GF P14 tissues. Genes encoding mucus-processing proteins (Clca1 and Tgm3) and antimicrobial factors (Itln1, Mptx1, Lgals3, and Tf) showed age-dependent increases but were unaffected by colonization, suggesting developmentally programmed IML maturation in ConvR mice (Fig. 2 D).

Next, we examined whether the early neonatal suppression of mucus growth by serine/cysteine proteases was microbiota regulated. Ex vivo analysis of adult GF colonic tissue showed increased mucus growth upon Comp PIC treatment or treatment with a cysteine protease–specific inhibitor (E64), and conventionalizing GF mice for four weeks restored insensitivity to E64, confirming microbiota-dependent regulation of proteolytic mucus suppression (Fig. 2 E).

Prior studies implicated TLR-MyD88 signaling in colonic barrier function (Bhinder et al., 2014; Larsson et al., 2012). Comparing adult *MyD88*^+/+^ and *MyD88*^−/−^ mice, we found no differences in IML thickness, barrier function, baseline mucus growth, or EDTA sensitivity, indicating MyD88 is not required for barrier properties or Clca1-mediated mucus expansion (Fig. 2, F–I). However, Comp PIC treatment in *MyD88*^−/−^ tissue increased mucus growth, resembling neonatal tissues, demonstrating that MyD88 negatively regulates cysteine protease–mediated mucus suppression (Fig. 2 I). Thus, MyD88 signaling does not regulate Clca1-mediated mucus expansion but is a negative regulator of cysteine protease mucus growth suppression, illustrating that these proteolytic processes are regulated independently from each other.

These findings show that postnatal IML maturation is driven by both microbiota-dependent and -independent mechanisms, with MyD88 signaling playing a key role in regulating proteolytic suppression of mucus expansion in early life.

### Microbiota colonization drives postnatal GC maturation

**Figure 3.**
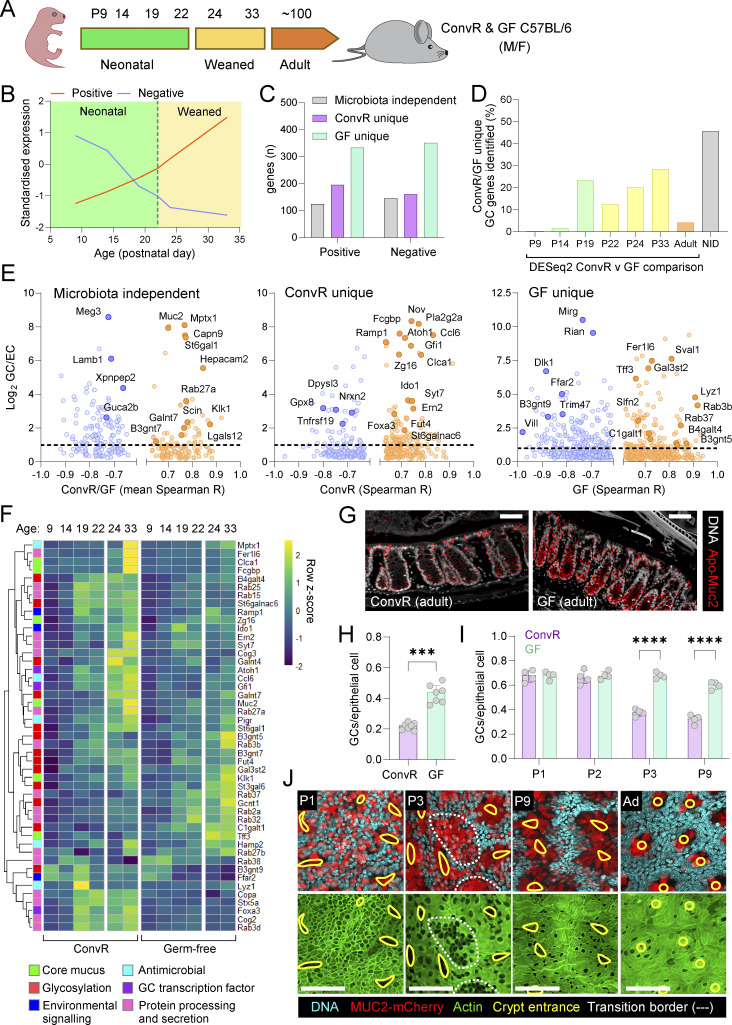
**Microbiota colonization and GC maturation. (A)** Sampling time points in postnatal days (P) of neonatal, weaned, and adult ConvR and GF mice. **(B)** Illustration of postnatal gene expression patterns with positive or negative monotonic correlation to age. **(C)** Total number of GC-enriched genes with positive or negative monotonic correlation to age in both ConvR and GF mice (microbiota independent) or in only ConvR or GF mice (microbiota dependent) was determined by Spearman correlation analysis of DESeq2 normalized RNA sequencing data. **(D)** Proportion of microbiota-dependent gene expression patterns shown in C identified by standalone DESeq2 comparison of ConvR and GF samples at individual ages. Genes not identified in any pairwise comparison are classified as “not identified” (NID). **(E)** Comparison of Spearman correlation coefficients (R) and GC expression enrichment (log_2_ GC:enterocyte [EC] expression ratios) of genes with significant positive (orange) or negative (blue) monotonic correlation to age. Plots show GC-enriched genes with microbiota-independent (left) or -dependent (middle, right) expression patterns. **(F)** Heatmap showing standardized expression (z-score) of selected GC-enriched genes with significant microbiota-dependent or -independent expression correlation to age. **(G)** Confocal micrographs of fixed colonic tissue from adult ConvR and GF mice stained for DNA (grey) and Apo-Muc2 (red). **(H)** Quantification of Apo-Muc2–positive cells as a fraction of total epithelial cells in adult ConvR and GF tissue based on images shown in G. **(I)** Quantification Apo-Muc2–positive cells as a fraction of total epithelial cells in P1–9 neonatal ConvR and GF tissues based on images shown in Fig. S1 C. **(J)** Whole-mount confocal imaging of neonatal and adult RedMUC2^98tr^ colon stained for DNA (blue), F-actin (green), and MUC2-mCherry (red). Crypt entrances (yellow line) and intercrypt transition zones between high and low GC density areas (white dashed line) are indicated. Images show x/y-axis maximum intensity projections. Data represent *n* = 2–4 (C–F), *n* = 4–7 (G–I), or *n* = 4 (J) animals per group, as indicated. All data are pooled from at least two independent litters or experiments. All error-bar graphs show median and interquartile range. Statistical comparisons between groups by Spearman correlation with Benjamini–Hochberg FDR correction (C–F), Mann–Whitney test (H), or two-way ANOVA and Fisher’s Least Significant Difference (LSD) (I); P < <0.001 (***), <0.0001 (****). Image scale bars are 50 µm.

**Figure S3.**
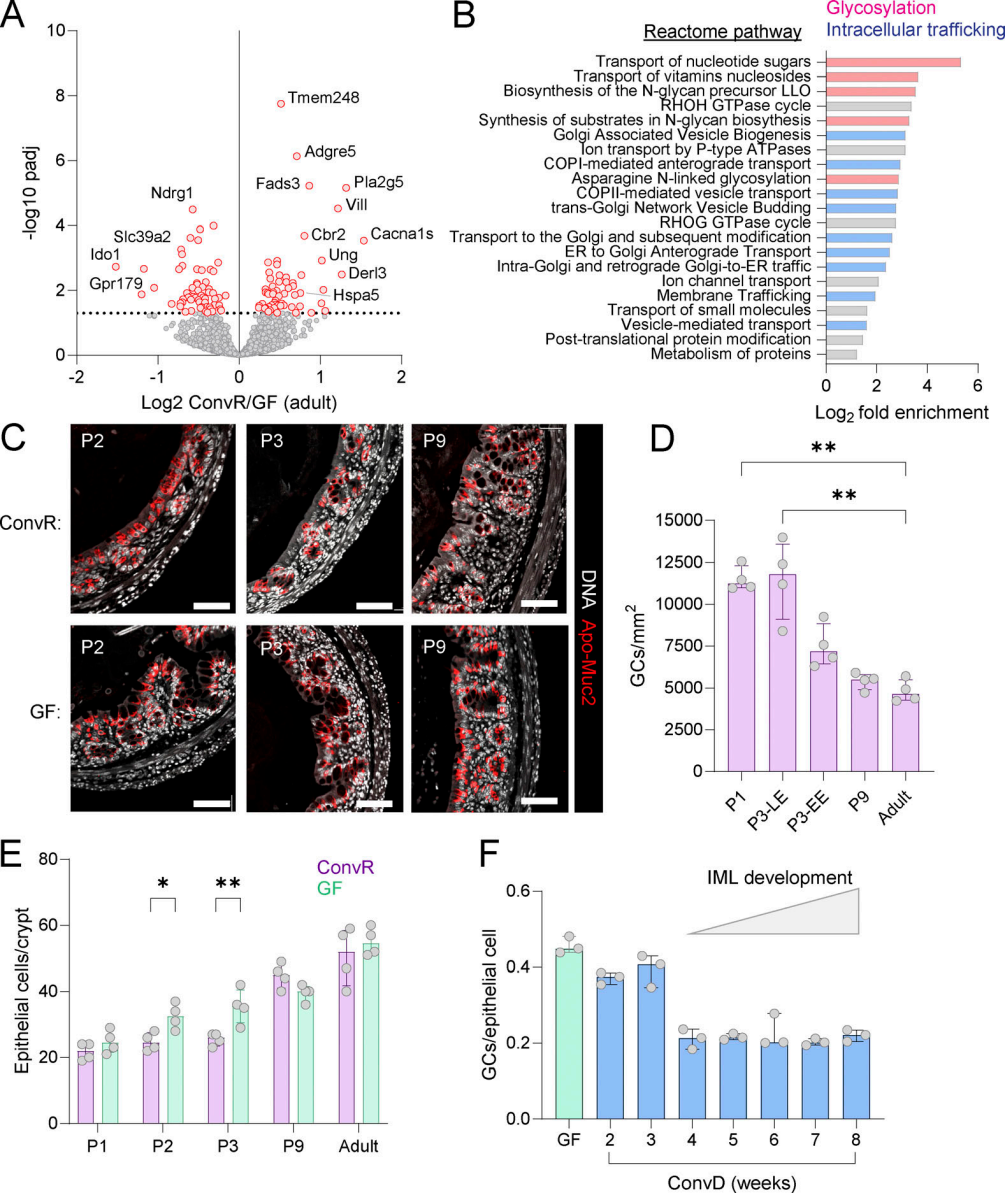
**R** **elated to** Fig. 3 **. (A)** Volcano plot of bulk mRNA sequencing data illustrating GC-enriched genes differentially expressed between ConvR and GF adult colon. **(B)** Reactome pathways significantly enriched in microbiota-regulated GC genes identified as positively correlated with postnatal age. **(C)** Confocal micrographs of fixed colonic tissue from adult ConvR and GF mice stained for DNA (grey) and Apo-Muc2 (red). **(D)** Quantification of mCherry^+^ GCs from colonic whole mounts based on images shown in Fig. 3 J. P3 samples are divided into late epithelium (LE) and early epithelium (EE). **(E)** Quantification of total epithelial cells/crypt in P1–9 neonatal and adult ConvR and GF tissues based on images shown in Fig. 3 G and Fig. S1 C. **(F)** Quantification of Apo-Muc2–positive cells as a fraction of total epithelial cells in adult GF and conventionalized (ConvD) mice at different time points after colonization. Data represent *n* = 4 (A–D) or *n* = 3 (D) animals per group. All error-bar graphs show median and interquartile range. All data are pooled from at least two independent litters or experiments. Statistical comparisons by one-way ANOVA and Fisher’s LSD; P < 0.05 (*), P < 0.01 (**). Image scale bars are 50 µm.

After investigating microbiota influences on IML maturation, we examined how age and microbial exposure shape colonic GC development. Bulk RNA sequencing of neonatal (P9–P22), weaned (P24–P33), and adult (>P100) ConvR and GF colonic tissues (Fig. 3 A) was filtered for 4098 GC-enriched genes (Nystrom et al., 2021). Differential expression analysis (DESeq2) identified microbial modulation of 120 GC-enriched genes in adults, including induction of ER chaperone BIP (*Hspa5*) and repression of *Ido1* (Fig. S3 A). While these differences were potentially relevant to GC function, only 12/120 genes had a log_2_ fold change ≥1, reflecting only modest microbiota-dependent mRNA expression differences in adult tissues.

We hypothesized that these minor adult differences might mask more significant microbiota-driven changes occurring postnatally. To capture age-dependent effects, we analyzed gene expression trends over P9–P33 by Spearman’s rank correlation, identifying 1,314 GC genes with age-correlated expression (illustrated in Fig. 3 B), 79% of which depended on microbiota colonization status (Fig. 3 C). Notably, 46% of these genes were undetected in pairwise ConvR vs. GF DESeq2 comparisons at individual time points (Fig. 3 D), highlighting the application of this approach in identifying microbial influences on GC gene expression.

Examining GC-enriched genes (Fig. 3, E and F), we found that *Muc2* expression correlated with postnatal age in both ConvR and GF mice. However, age-dependent induction of other core mucus components was microbiota-specific: *Clca1*, *Fcgbp*, and *Zg16* were induced in ConvR mice, while *Tff3* was induced in GF mice. Secretory transcription factors (*Atoh1*, *Gfi1*, and *Foxa3*) were positively correlated with age only in ConvR mice, suggesting microbiota-driven GC maturation. Functional Reactome pathway enrichment analysis confirmed microbiota-dependent regulation of genes linked to mucin glycosylation (e.g., *Slc35b3*, *Slc35a2*, *Galnt4*, *Fut4*, and *C1galt1*) and intracellular cargo transport (*Stx5a*, *Copa*, and *Cog2*) (Fig. S3 B).

We next investigated the possibility that microbiota-dependent postnatal induction of GC gene expression was reflective of increased GC numbers, as it is frequently claimed that GF mice have fewer intestinal GCs than ConvR animals (Deplancke and Gaskins, 2001). We quantified GCs using an intracellular marker by staining for the immature ER form of Muc2 (Apo-Muc2). Surprisingly, adult GF mice had higher GC frequency than ConvR mice (Fig. 3, G and H). In early neonates (P1–2), both groups had similar GC numbers, but by P3, GC frequency declined in ConvR mice, stabilizing at adult levels (Fig. 3 I and Fig. S3 C). Whole-mount imaging of colonic tissue from RedMUC2^98tr^ Muc2 reporter mice confirmed this transition, showing a shift from GC-rich late epithelial intercrypt regions to early epithelial cells developing from crypts with fewer GCs (Fig. 3 J and Fig. S3 D). Assessing total epithelial cell counts, we found no GC reduction due to expansion of other epithelial cell types, as P2–P3 GF mice had slightly higher epithelial cell numbers than ConvR neonates (Fig. S3 E). Lastly, examination of archived tissues from adult GF mice conventionalized by fecal transfer from ConvR mice identified a similar GC reduction after 3–4 wk, immediately prior to the period of IML normalization (Johansson et al., 2015) (Fig. S3 F).

These findings demonstrate that microbiota colonization plays a crucial role in postnatal GC maturation, reducing total GC frequency while inducing transcriptional programs associated with secretory function and mucin glycosylation.

### Microbiota colonization drives postnatal maturation of colonic senGCs

**Figure 4.**
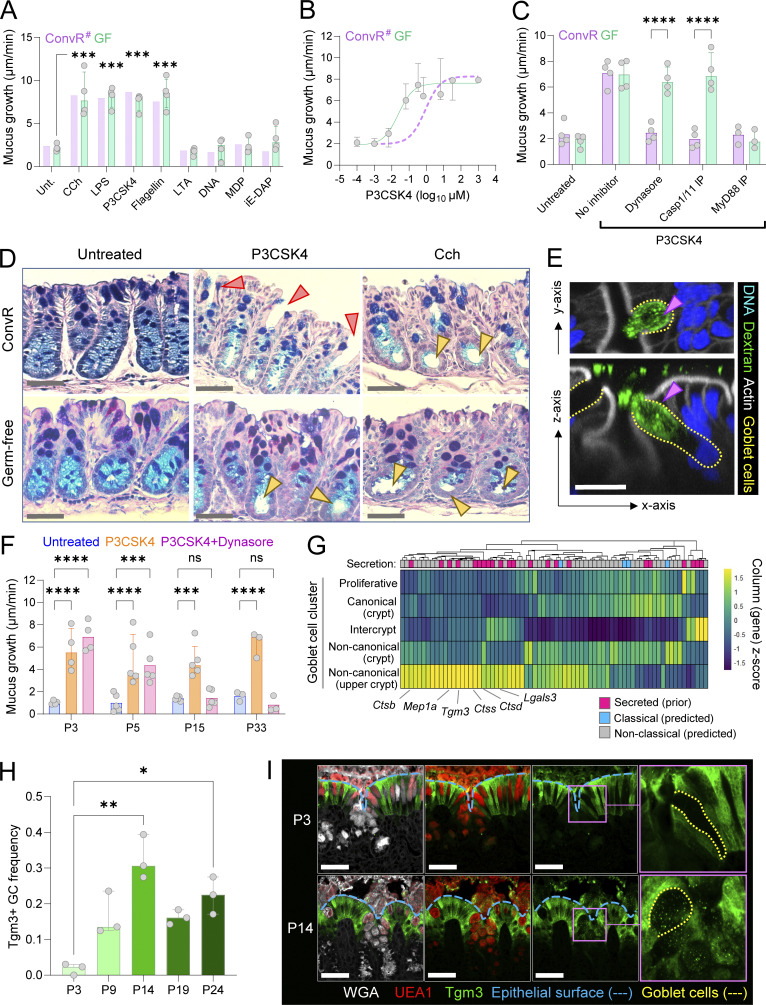
**senGC maturation is microbiota dependent. (A)** Ex vivo mucus growth in adult GF and ConvR mouse colon after stimulation with bacterial MAMPs. **(B)** Ex vivo mucus growth dose response to P3CSK4 in adult GF and ConvR mouse colon. **(C)** Ex vivo mucus growth in adult GF and ConvR mouse colon stimulated with P3CSK4 in the presence or absence of senGC activation inhibitors. **(D)** AB/PAS-stained tissue sections from ex vivo experiments illustrated in A. Emptied upper crypt GCs (red arrowheads) and lower crypt cavitation (yellow arrowheads) indicated. **(E)** Whole-mount confocal imaging of adult GF mouse colon treated with fluorescent dextran tracer. Images show x/y-axis (upper panel) and x/z-axis (lower panel) cross-sections illustrating dextran uptake by an upper crypt GC (purple arrowhead). **(F)** Ex vivo mucus growth in neonatal (P3, 5, and 15) and postweaning (P33) rat colon stimulated with P3CSK4 in the presence or absence of Dynasore inhibitor. **(G)** Standardized expression of genes (columns) encoding known and predicted secreted proteins upregulated in mucus from P9-adult compared with P1–P7 rats (see Fig. 1 H) in GC subpopulations (rows) identified by scRNA-seq. “Secretion” row indicates evidence of secretion determined by prior annotation or in silico predication of classical or nonclassical secretion by SecretomeP. **(H)** Quantification of the frequency of Tgm3-expressing GCs as a proportion of the total GC population in neonatal (P3, P9, P14, and P19) and postweaning (P24) colonic tissue sections from ConvR mice. **(I)** Confocal micrographs of representative tissue sections from P3 and P14 ConvR mice stained for Tgm3 (green) or the epithelial border and GC-binding lectin WGA (grey) and the GC-specific lectin UEA1 (red). The epithelial surface (blue dashed line) and an individual GC from each image is indicated (yellow dashed line). Data represent *n* = 3–5 (A–F, H, and I) animals per group, as indicated. All data are pooled from at least two independent litters or experiments. All error-bar graphs show median and interquartile range. Statistical comparisons between groups by two-way ANOVA and Fisher’s LSD (A and C) or Kruskall–Wallis and uncorrected Dunn’s test (F and H); P < 0.05 (*), <0.01 (**), <0.001 (***), <0.0001 (****). Image scale bars are 50 µm (D and I) or 20 µm (E). # note: ConvR data displayed in A and B are reproduced from our previous publication (Birchenough et al., 2016) and are shown for illustrative purposes only.

After examining microbiota influences on GC maturation on a bulk tissue level, we specifically investigated the functional development of senGCs, a GC subpopulation involved in inflammasome-dependent mucus secretion from upper crypt GCs in response to bacterial microbe-associated molecular patterns (MAMPs) (Birchenough et al., 2016). Ex vivo analysis of GF colonic tissues revealed that mucus secretion in response to bacterial MAMPs (P3CSK4, LPS, and flagellin) was similar to responses previously observed in ConvR tissues (Fig. 4 A). However, GF tissues were ∼36 times more sensitive to P3CSK4 than ConvR tissues, suggesting divergent secretory mechanisms (Fig. 4 B).

**Figure S4.**
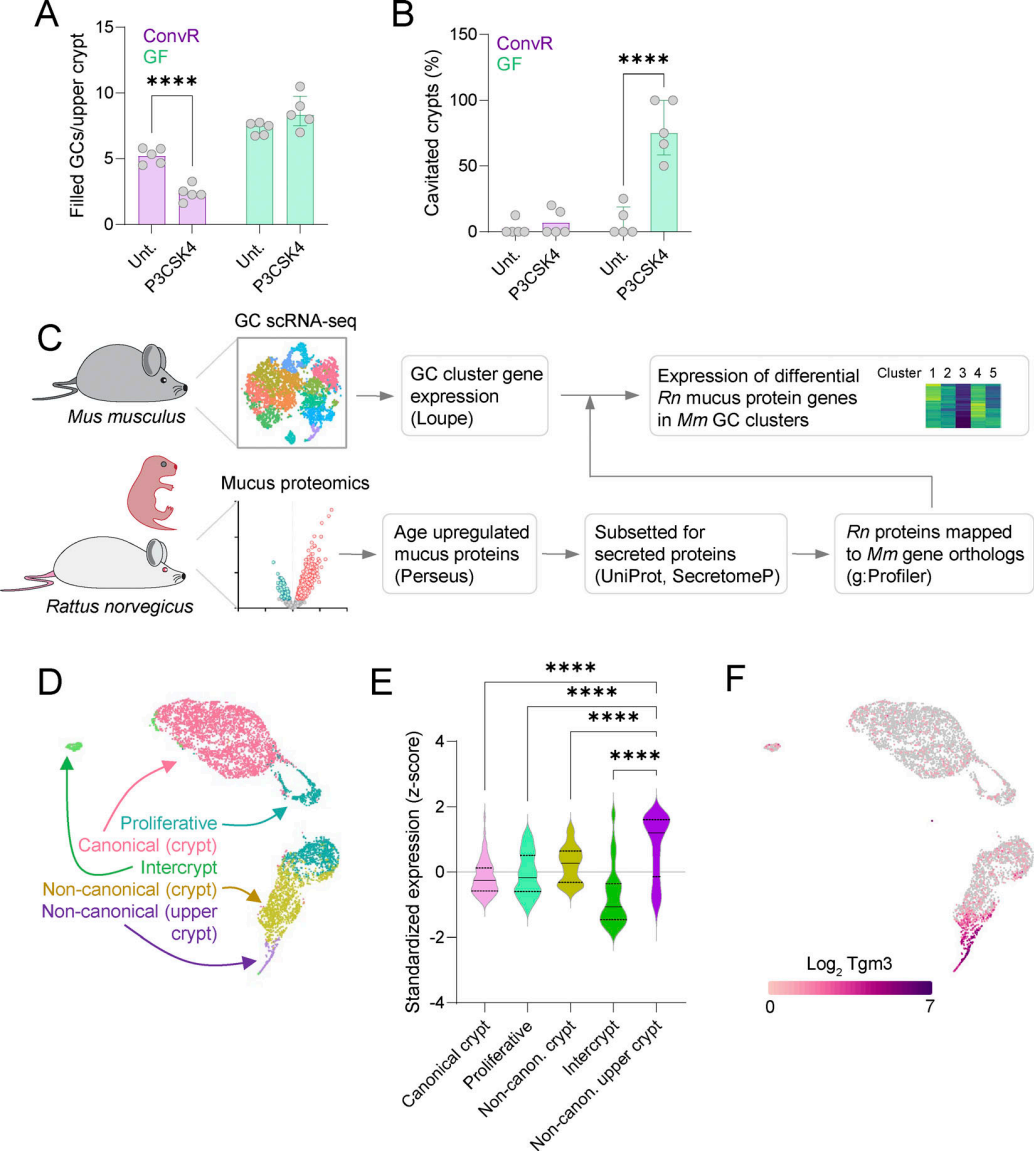
**R** **elated to** Fig. 4 **. (A)** Filled upper crypt GCs/crypt quantified from AB/PAS-stained tissue sections shown in Fig. 4 D. **(B)** Percentage of cavitated crypt bases quantified from AB/PAS-stained tissue sections shown in Fig. 4 D. **(C)** Processing pipeline for determining the expression of age-regulated mucus protein genes (see Fig. 1 H) in GC clusters identified by scRNA-seq. Databases and software used are shown in parentheses. *Rn*, *R. norvegicus*;* Mm*, *M. musculus. ***(D)** UMAP plot of scRNA-seq data from isolated colonic GCs highlighting previously annotated GC clusters (Nystrom et al., 2021). **(E)** Per GC cluster standardized gene expression (z-scores) of secreted proteins (*n* = 72) identified as significantly enriched in the P9-Adult compared with P1–P7 colonic mucus proteome (see Fig. 1, G and H; and Fig. 4 G). **(F)** UMAP plot illustrating log_2_ expression of *Tgm3* in colonic GC scRNA-seq data. Data from *n* = 5 replicates (A and B) or aggregated from *n* = 2 independent experiments (D–F). Graphs show median and interquartile range. Statistical comparisons between clusters by two-way (A and B) or one-way (E) ANOVA and Fisher’s LSD; P < 0.0001 (****).

To determine if GF MAMP-induced secretion followed the senGC-dependent pathway, we treated GF and ConvR tissues with P3CSK4 alongside inhibitors targeting elements of the senGC activation pathway: endocytosis (Dynasore), inflammasome activation (Caspase 1/11 inhibitor), and MyD88 signaling. Mucus secretion from GF tissues remained MyD88-dependent; however, unlike ConvR tissues, GF mucus secretion was unaffected by endocytosis and inflammasome inhibition, indicating a senGC-independent pathway (Fig. 4 C). Histological analysis of AB/PAS-stained sections of tissue collected after treatment supported this, showing upper crypt GC emptying in ConvR tissues upon P3CSK4 treatment, while in GF tissues, secretion occurred only in lower crypt GCs (Fig. 4 D; and Fig. S4, A and B). Despite not mounting an endocytosis-dependent secretory response, confocal imaging of GF colonic whole mounts exposed to fluorescent dextran confirmed that GF upper crypt GCs were capable of endocytosis, despite the absence of functional senGCs (Fig. 4 E).

Next, we assessed the postnatal development of the senGC response in ConvR neonatal rats. Ex vivo mucus growth assays showed that P3 and P5 rats exhibited the senGC-independent secretory mode observed in adult GF mice, whereas P15 and older tissues transitioned to senGC-dependent secretion, indicating age-dependent senGC priming in the pre-weaning period (Fig. 4 F).

Since senGC maturation closely coincided with postnatal changes in the mucus proteome (Fig. 1, G and H), we screened for a senGC signature in our mucus proteomic data. Mucus proteins with increased abundance over the senGC developmental period were filtered based on validated (e.g., UniProt annotation) or predicted (in silico SecretomeP analysis) secretion and mapped onto GC subpopulations previously annotated after single-cell RNA sequencing (scRNA-seq) analysis (Nystrom et al., 2021) (Fig. S4, C and D). This prior study had identified expression of senGC activation genes (e.g., *Nlrp6*) in upper crypt noncanonical GCs in mice (Nystrom et al., 2021), and comparing mucus proteins upregulated in P9-adult rats to gene expression in different GC clusters (Fig. S4 D) identified an association between increased postnatal mucus protein abundance and noncanonical upper crypt GCs, particularly Tgm3 and various proteases (Ctss, Ctsb, Ctsd, and Mep1a) (Fig. 4 G and Fig. S4 E). Within the GC population, Tgm3 expression served as a specific marker of noncanonical upper crypt GCs (Fig. S4 F), and tracking Tgm3 expression in ConvR mice by immunostaining revealed a rise in Tgm3^+^ GCs from negligible levels at P3 to ∼32% at P14, decreasing to ∼22% after weaning (Fig. 4, H and I).

These findings demonstrate that functional senGCs develop in the postnatal period under microbiota regulation and that maturation of the senGC secretory response is linked to the emergence of the upper crypt noncanonical GC lineage, previously associated with senGC function.

### senGC maturation requires microbiota colonization

**Figure 5.**
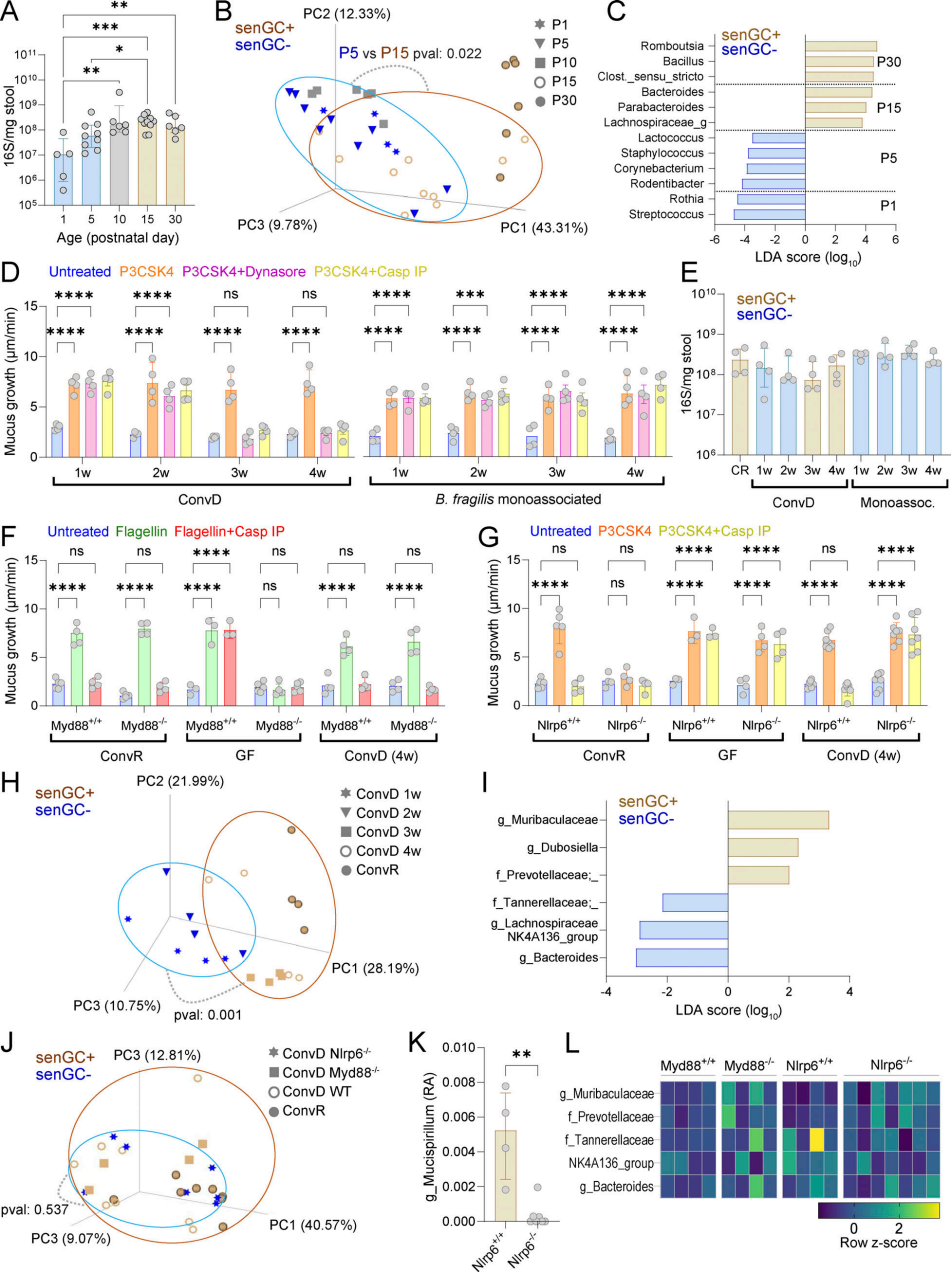
**Microbiota-dependent induction of senGC function. (A)** Total bacterial load in the colon of rats at different postnatal days quantified by 16S qPCR of stool DNA. **(B)** Principal coordinate analysis of postnatal rat microbiota beta diversity (unweighted UNIFRAC) based on metataxonomic 16S sequencing of DNA from stool samples. **(C)** Linear discriminant analysis (LDA) size effect analysis of bacterial taxa significantly enriched in stool from rats at different ages. Taxa enrichment in specific age groups is indicated. **(D)** Ex vivo mucus growth in adult conventionalized (ConvD) and *B. fragilis* monoassociated mouse colon stimulated with P3CSK4 in the presence or absence of senGC activation inhibitors targeting endocytosis (Dynasore) or inflammasome activation (Casp IP). **(E)** Total bacterial load in colon of conventionally raised (CR), ConvD, and monoassociated mice quantified by 16S qPCR of stool DNA. **(F)** Ex vivo mucus growth in adult *MyD88*^+/+^ and *MyD88*^−/−^ ConvR, GF, and 4-wk (w) ConvD mouse colon stimulated with flagellin in the presence or absence of a senGC activation inhibitor targeting inflammasome activation (Casp IP). **(G)** Ex vivo mucus growth in adult *Nlrp6*^+/+^ and *Nlrp6*^−/−^ ConvR, GF, and 4-wk ConvD mouse colon stimulated with P3CKS4 in the presence or absence of a senGC activation inhibitor targeting inflammasome activation (Casp IP). **(H)** Principal coordinate analysis of microbiota beta diversity (Bray–Curtis dissimilarity) based on metataxonomic 16S sequencing of DNA from ConvR and ConvD stool samples. **(I)** Linear discriminant size effect analysis of bacterial taxa significantly enriched in stool from mice with the senGC^−^ or senGC^+^ phenotype. **(J)** Principal coordinate analysis of microbiota beta diversity (Bray–Curtis dissimilarity) based on metataxonomic 16S sequencing of DNA from ConvR WT, ConvD WT, and ConvD *MyD88*^−/−^ and *Nlrp6*^−/−^ stool samples. **(K)** Relative abundance (RA) of the genus *Mucispirillum* in ConvD *Nlrp6*^+/+^ and *Nlrp6*^−/−^ mice determined by metataxonomic 16S sequencing of stool DNA. **(L)** Standardized abundance (z-score) of bacterial taxa identified in F in 16S sequencing data from ConvD WT and ConvD *MyD88*^−/−^ and *Nlrp6*^−/−^ stool samples. Data represent *n* = 4–9 animals per group, as indicated. All data are pooled from at least two independent experiments or litters. Where relevant (A–C, E, and H–K) experimental groups are color coded by the absence (senGC^−^; blue) or presence (senGC^+^; brown) of the senGC-dependent secretory response. All error-bar graphs show median and interquartile range. Statistical comparisons between groups by two-way ANOVA and Fisher’s LSD (D, F, and G), Kruskal–Wallis and Dunn’s multiple comparison (A and E), PERMANOVA (B, H, and J), or Mann–Whitney test (K); P < 0.05 (*), <0.01 (**), <0.001 (***), <0.0001 (****).

To assess microbiota dependency in senGC maturation, we analyzed microbiota dynamics in rat stool samples over P1–P30, the period encompassing senGC functional development. 16S qPCR and beta diversity analysis after 16S DNA sequencing (unweighted unique fraction metric [UNIFRAC]) showed significant shifts in bacterial load and community structure between P5–P15, coinciding with senGC maturation (Fig. 5, A and B). Analysis of age-discriminative taxa by linear discriminant analysis effect size (LEfSe) identified several that were significantly enriched in rats at P15 and older (Fig. 5 C), with the most abundant (average 5.8% of total 16S reads) being the genus *Bacteroides*.

To test whether microbial exposure could induce senGC maturation in adults, we conventionalized GF mice via full microbiota transfer (ConvD) or monoassociated them with a representative of the *Bacteroides* genus (*Bacteroides fragilis*) to examine the effect of exposure to a single bacterial species. We detected development of a MAMP (P3CSK4)-induced senGC-dependent response after 3 wk in ConvD mice; however, monoassociated mice failed to elaborate this response at any of the time points tested, despite comparable bacterial loads (Fig. 5, D and E). This indicated that a set of microbial signals beyond simple detection of common bacterial MAMPs by innate immune signaling mechanisms are likely required to drive functional senGC maturation.

To confirm that major innate immune pathways were not involved, we conventionalized GF *MyD88*^−/−^ and *Nlrp6*^−/−^ mice and assessed their secretory response at 4 wk in comparison to ConvR and GF mice of the same genotypes. Using flagellin as a MyD88-independent senGC activator (Birchenough et al., 2016), we found that while MyD88 was critical for senGC-independent (GF) secretion, it was not required for senGC maturation, as ConvD *MyD88*^*−/−*^ secretory responses phenocopied ConvR mice (Fig. 5 F). Conversely, application of a similar analytical approach to *Nlrp6*^−/−^ mice confirmed that Nlrp6 inflammasome function was only required for the senGC-dependent secretory response in ConvR mice; however, surprisingly, ConvD *Nlrp6*^−/−^ mice maintained the senGC-independent secretory response after colonization (Fig. 5 G). This result could suggest that Nlrp6 is important for both the maturation and activation of senGCs; however, it should be noted that ConvR *Nlrp6*^−/−^ mice do not maintain senGC-independent secretory response. This indicated that aspects of adult GF conventionalization that are divergent in *Nlrp6*^+/+^ and *Nlrp6*^−/−^ mice may play a role in the inability of ConvD *Nlrp6*^−/−^ animals to switch from the senGC-independent to the senGC-dependent MAMP-induced secretory response.

Previous studies have indicated that conventionalization of adult GF *Nlrp6*^−/−^ mice results in an altered microbiota configuration (Levy et al., 2015). We hypothesized that variable senGC status in ConvD mice was linked to differences in microbiota composition. Beta diversity analysis (Bray–Curtis dissimilarity) of gut microbiota composition after 16S DNA sequencing showed significant differences between the early stage (1–2 wk, senGC-negative) and late-stage (3–4 wk, senGC-positive) ConvD mice (Fig. 5 H). LEfSe identified taxa linked to senGC maturation, including *Muribaculaceae* and *Dubosiella* (positively associated) and *Bacteroides* and *Lachnospiraceae* (negatively associated) (Fig. 5 I). To help identify if these differences were might be causal, we compared the microbiota configurations between 4-wk-old ConvD WT and MyD88^−/−^ mice (senGC positive) with those in 4-wk-old ConvD Nlrp6^−/−^ mice (senGC negative) to determine if alterations in these bacterial taxa were consistently correlated with senGC function. In this case, we did not detect any differences in overall microbiota composition between these groups (Fig. 5 J). Pairwise analysis of ConvD *Nlrp6*^+/+^ and *Nlpr6*^−/−^ mice did identify some differences in minor bacterial taxa, notably the genus *Mucispirillum* (Fig. 5 K). However, none of the bacterial taxa that were correlated with senGC maturation in our previous analysis were altered between senGC-positive or senGC-negative mice under these conditions (Fig. 5 L), thus indicating that such correlations were unlikely to be causal.

Combined, these results demonstrated that maturation of the senGC-dependent secretory response was associated with colonic microbiota alterations and inducible by full microbiota colonization, but that senGC maturation could not be initiated by exposure to a single bacterium (*B. fragilis*) or by detection of bacterial MAMPs via MyD88 signaling.

### Microbiota-induced senGC maturation coincides with increased protection from colitogenic challenge

The senGC subpopulation is thought to play a role in preventing inflammation by protecting the colonic crypts from bacteria that breach the IML barrier. Microbiota-induced senGC maturation therefore presents an opportunity to study the impact of senGC proficiency or deficiency without the need for genetic modifications that risk off-target effects. Furthermore, by inducing senGC maturation in adult GF mice through controlled colonization, we can isolate its effects from other postnatal developmental factors and apply colitis challenge models not feasible in neonates.

**Figure 6.**
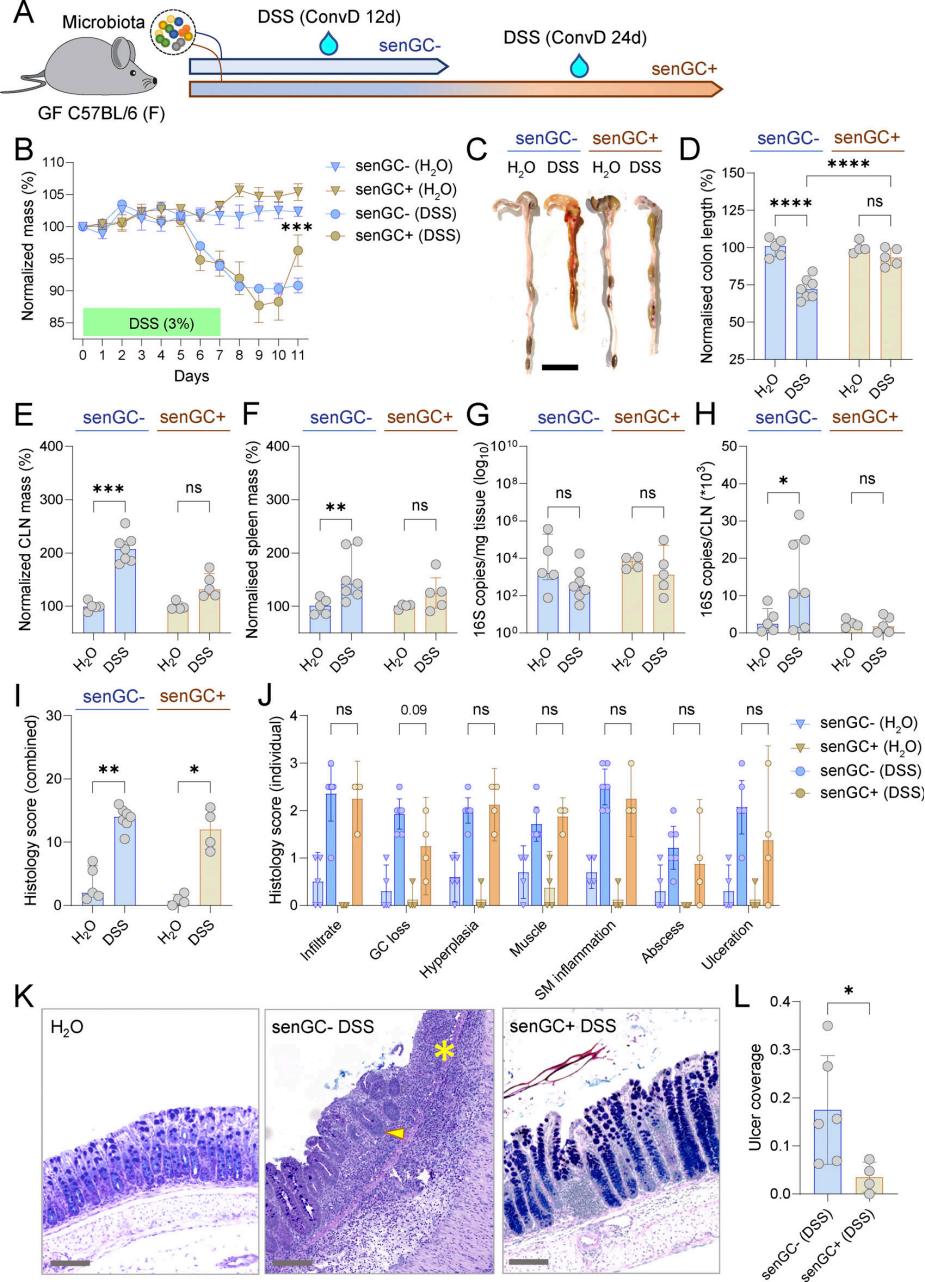
**Induction of senGC maturation coincides with colitis protection. (A)** Timing of colitogenic DSS challenge in relation to microbiota-dependent induction of senGC maturation in adult GF mice. **(B)** Tracking of day zero normalized mass changes in H_2_O- and DSS-treated ConvD mice from senGC^−^ and senGC^+^ groups. **(C)** Photographs of colonic tissue dissected from different treatment groups at the termination of the DSS challenge experiment. **(D)** Quantification of total colon length in different treatment groups based on images shown in C. All data normalized to average colon length of the H_2_O control animals for each group. **(E)** Quantification of CLN mass in different treatment groups. All data normalized to average CLN mass of the H_2_O control animals for each group. **(F)** Quantification of spleen mass in different treatment groups. All data normalized to the average spleen mass of the H_2_O control animals for each group. **(G)** Determination of mucosal bacterial load in different treatment groups by 16S qPCR of colonic tissue DNA. **(H)** Determination of bacterial translocation from the colon in different treatment groups by 16S qPCR of CLN DNA. **(I)** Combined histology scores from different treatment groups determined by histopathological scoring of fixed colonic Swiss roll tissue sections. **(J)** Individual scores for different histopathology metrics from different treatment groups. **(K)** Micrographs of AB-PAS–stained fixed colonic Swiss roll tissue sections used for histology scoring. Areas of ulceration (asterisk) and GC depletion (yellow arrowhead) in senGC^−^ DSS-treated tissue are indicated. **(L)** Ulcer coverage in DSS-treated mice as a fraction of total mucosal surface area. Data represent *n* = 4–7 animals per group, as indicated. All data are pooled from two independent experiments. All error-bar graphs show median and interquartile range. Statistical comparisons between groups by Kruskal–Wallis and uncorrected Dunn’s (B–J) or Mann–Whitney test (L); P < 0.05 (*), <0.01 (**), <0.001 (***), <0.0001 (****). Image scale bars are 2 cm (C) or 100 µm (K).

To test senGC function in colitis, adult GF mice were conventionalized for either 12 days (senGC negative) or 24 days (senGC positive) before exposure to dextran sodium sulfate (DSS) (Fig. 6 A). The senGC status of challenged mice was confirmed by lack of the senGC-dependent secretory response in littermate mice conventionalized in parallel (Fig. 5 D). Mice received 3% DSS for 7 days, followed by a 4-day recovery period. Both groups experienced similar weight loss during DSS treatment, but senGC-positive mice showed faster recovery (Fig. 6 B). In addition, colonic and lymphatic tissue analysis revealed greater reductions in colon length and increased caudal LN (CLN) and spleen mass in DSS-treated senGC-negative mice (Fig. 6, C–E). 16S qPCR detected comparable bacterial loads in colonic mucosa of both groups (Fig. 6 G); however, we found a higher bacterial burden in CLNs of senGC-negative mice, suggesting increased bacterial translocation (Fig. 6 H).

Lastly, we assessed colon tissue histopathology by scoring colonic Swiss roll tissue sections for inflammatory infiltrate, GC loss, epithelial hyperplasia, muscle thickening, submucosal inflammation, and the development of mucosal abscesses and ulceration (Fig. 6, I–K). Overall, we did not observe any differences between the combined histology scores of the senGC-negative and senGC-positive DSS-treated groups (Fig. 6 I); however, individual elements of the pathology index showed a tendency (albeit nonsignificant) toward lower scores related to GC loss and ulceration in the senGC-positive DSS-treated mice (Fig. 6, J and K). In support of this indicative data, the proportion of ulcerated colonic epithelium was significantly higher in the senGC-negative DSS-treated mice (Fig. 6 L).

**Figure S5.**
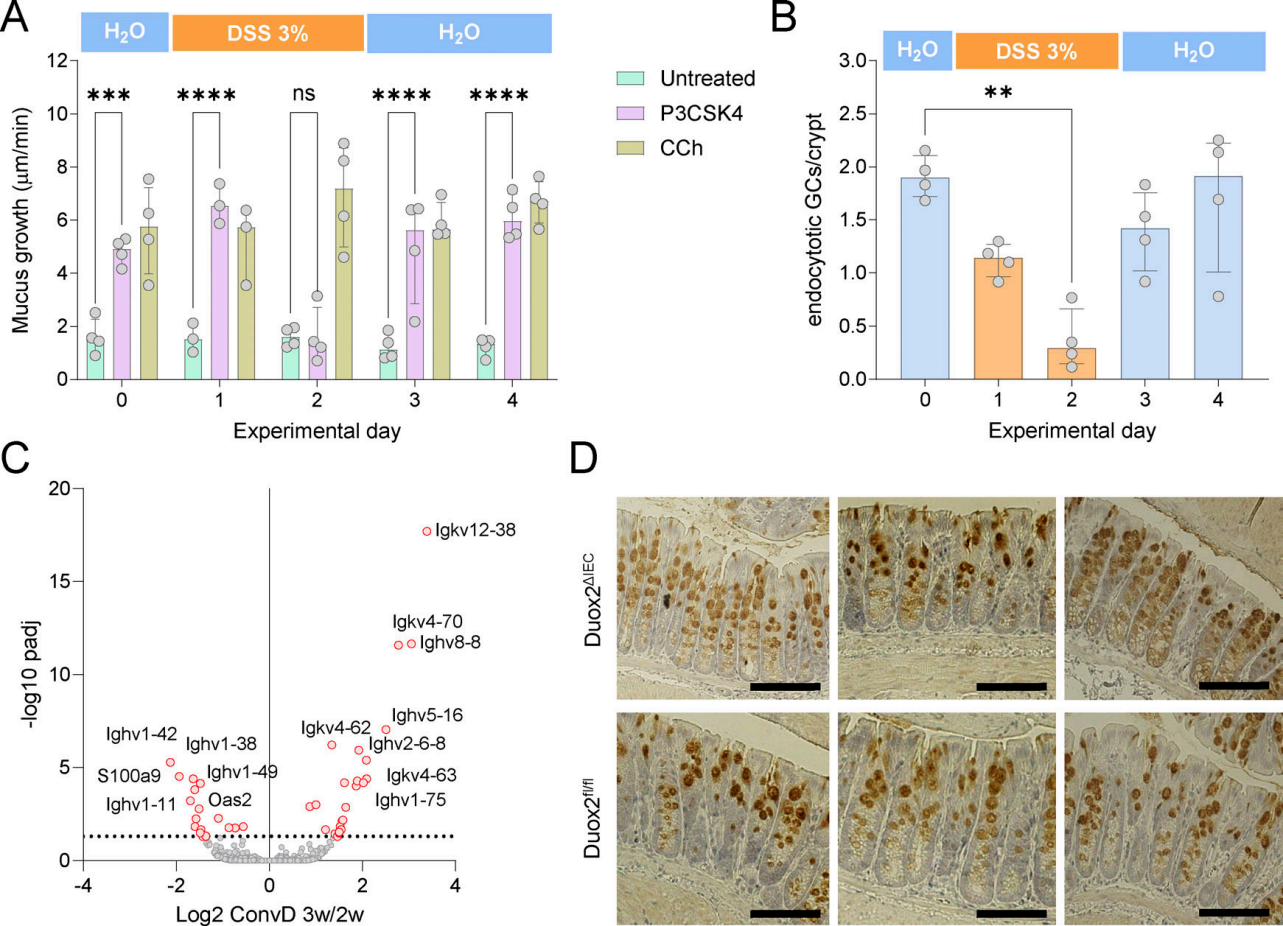
**R** **elated to** Figs. 6 and 7**. (A)** Quantification of senGC-dependent secretion in mice prior (day [d]0), during (d1–2), or after (d3–4) exposure to DSS in drinking water by ex vivo mucus growth stimulated with P3CSK4 or carbachol (CCh). **(B)** Quantification of endocytotic GCs in fixed colon whole mounts of the same mice analyzed in A. **(C)** Volcano plot of bulk mRNA sequencing data illustrating differential gene expression between ConvD 3-wk (w) and ConvD 2w colonic tissue. **(D)** Representative micrographs of colonic tissue sections from Duox2^fl/fl^ and Duox2^ΔIEC^ mice after immunohistochemical staining for Muc2. Data represent *n* = 3–4 animals per group. All data are pooled from at two independent experiments. Statistical analysis by two-way (A) or one-way (B) ANOVA and Fisher’s LSD test; P < 0.01 (**), P < 0.001 (***), P < 0.0001 (****). Image scale bars are 100 µm.

We considered that reduced GC loss in senGC^+^ mice may be the result of intrinsic senGC resistance to DSS. However, quantification of senGC function and number after DSS exposure in ConvR mice indicated that senGCs are depleted within 48 h of colitogenic challenge (Fig. S5, A and B). As senGCs are normally depleted from the epithelium downstream of mucus secretion via Nlrp6 inflammasome activation (Birchenough et al., 2016), this finding implies that reduced GC loss in senGC^+^ mice may be linked instead to senGC protective functions. Lastly, we assessed transcriptomic differences between ConvD 2-wk and ConvD 3-wk mice that might explain altered DSS sensitivity. Bulk RNA sequencing from colonic tissues of both groups identified a small number of genes differentially regulated, with the majority (31/40 with Padj < 0.05) representing IgG genes that increased or decreased in expression over this colonization period (Fig. S5 C).

Our findings suggest that DSS treatment induces severe colitis in both senGC-negative and senGC-positive mice; however, microbiota-dependent senGC induction coincides with somewhat increased protection from GC depletion, ulceration, and microbiota breach of the mucosal barrier during DSS-mediated colitis. Our experiments do not rule out the possibility that other factors may contribute to these phenotypes; thus, it is not possible to definitively link a causal relationship to senGC maturation. However, given the proposed in vivo function of senGCs, these findings may provide further support to this protective mechanism.

### Microbiota colonization matures senGC responses via regulation of Duox2

**Figure 7.**
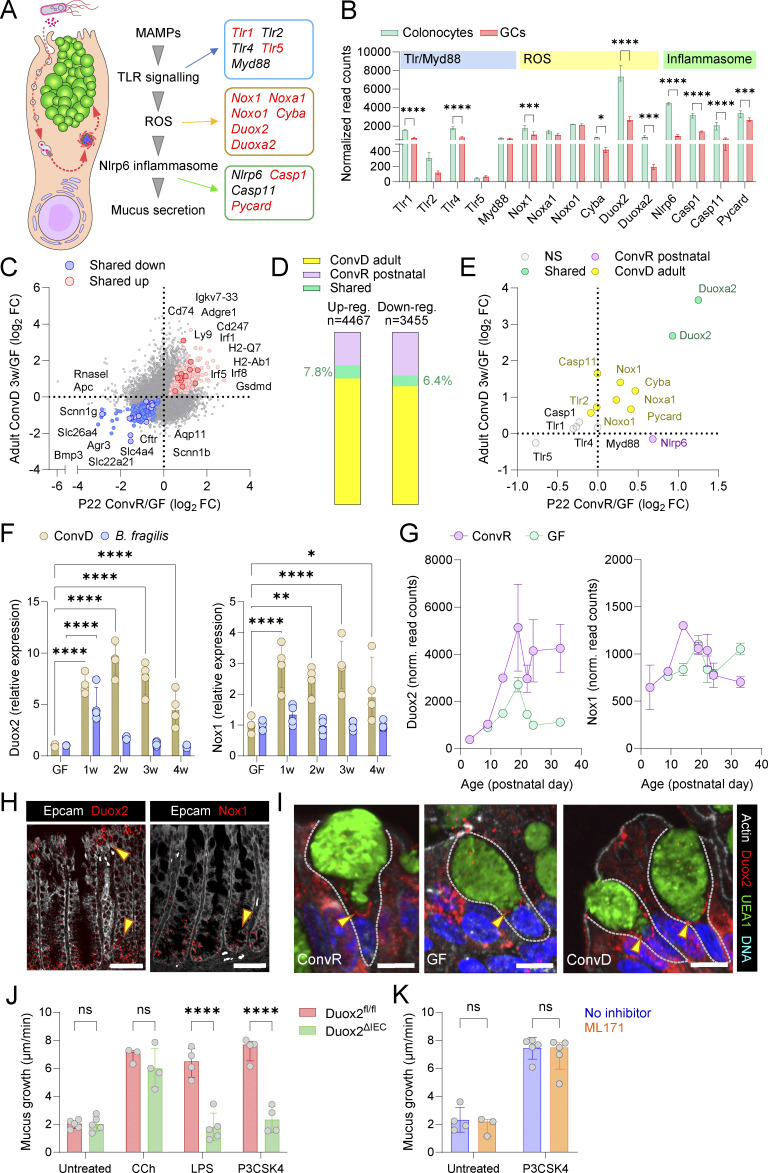
**Microbiota induction of senGC maturation via regulation of Duox2. (A)** Schematic of the senGC activation pathway highlighting known (black) and putative (red) pathway genes. **(B)** Expression of known and putative senGC genes in FACS-isolated colonic GCs and colonocytes determined by DESeq2 analysis of bulk RNA sequencing data. **(C)** Comparison of gene expression ratios between P22 ConvR:GF mice and adult 3-wk ConvD:GF mice quantified by DESeq2 analysis of bulk colonic RNA sequencing data. Genes significantly upregulated (red) or downregulated (blue) by microbiota exposure in both P22 and ConvD mice are indicated. **(D)** Proportion of unique and shared genes significantly regulated by microbiota exposure in P22 ConvR and adult 3-wk ConvD mice, based on data shown in C. **(E)** Comparison of microbiota-dependent expression of known and putative senGC activation pathway genes (A and B) in P22 ConvR and adult 3-wk ConvD mice. Subset of data shown in C. Genes not significantly regulated by microbiota in either group (grey) or genes regulated in either P22 ConvR (purple), adult ConvD (yellow), or both groups (teal) are indicated. **(F)** Relative expression (compared with GF) of *Duox2* (left) and *Nox1* (right) genes in ConvD (brown) and *B. fragilis* monoassociated (blue) mice from 1 to 4 wk (w) colonization. Expression determined by qRT-PCR of colonic RNA, normalized to *Gapdh* and *Rplp0* expression. **(G)** Expression of *Duox2* (left) and *Nox1* (right) genes in postnatal ConvR (purple; P3–33) and GF (teal; P9–P33) determined by DESeq2 analysis of bulk colonic RNA sequencing data. **(H)** Confocal micrographs of fixed colonic tissue sections from ConvR WT mice stained for *Duox2* (left) and *Nox1* (right) mRNA by in situ RNA hybridization and counterstained by Epcam (grey). Duox2- or Nox1-expressing crypt regions are indicated (yellow arrowheads). **(I)** Confocal micrographs showing upper crypt GCs in fixed colonic tissue sections from ConvR, GF, and ConvD mice stained for Duox2 (red), mucus (UEA1; green), actin (grey), and DNA (blue). Intracellular Duox2 in GCs are indicated (yellow arrowheads). **(J)** Ex vivo mucus growth in *Duox2*^fl/fl^ and *Duox2*^ΔIEC^ colon tissue treated with carbachol (CCh), LPS, or P3CSK4. **(K)** Ex vivo mucus growth in WT colon tissue treated with P3CSK4 in the presence or absence of the Nox1 inhibitor ML171. Data represent *n* = 2–5 animals per group, as indicated. All data are pooled from at least two independent experiments or litters. All error-bar graphs show median and interquartile range. Statistical comparisons between groups by DESeq2 (B, C, and E), Kruskal–Wallis and Dunn’s multiple comparison (F), or two-way ANOVA and Fisher’s LSD (J and K); P < 0.05 (*), <0.01 (**), <0.001 (***), <0.0001 (****). Scale bars are 50 µm (H) or 5 µm (I). FC, fold change.

Previous studies of senGC activation in response to MAMPs indicate that a GC-intrinsic sequence of endocytosis, TLR-MyD88 signaling, ROS synthesis, and Nlrp6 inflammasome assembly occurs upstream of mucus secretion (Fig. 7 A). Notably, analysis of RNA sequencing data from sorted GCs and colonocytes demonstrates that none of the confirmed or putative activation mediators are specifically expressed in GCs; on the contrary, most show higher expression levels in colonocytes (Fig. 7 B). We therefore examined total bulk mRNA sequencing data from both 3-wk ConvD adult mice and P22 postnatal ConvR mice and compared them to their age-matched GF controls to establish if microbiota-induced expression of senGC pathway genes might indicate a common mechanism of senGC maturation (Fig. 7 C). While microbiota-induced gene expression in adults and neonates overlapped in immune-related genes, only 7.8% of induced and 6.4% of suppressed genes were commonly regulated (Fig. 7 D). Notably, senGC pathway genes were either unchanged or selectively induced in ConvD adults (*Nox1*) or postnatal ConvR mice (*Nlrp6*). However, *Duox2* and its accessory *Duoxa2* were strongly upregulated in both models, suggesting a role in senGC maturation (Fig. 7 E).

We next examined evidence that regulation of Duox2 might provide a mechanistic basis for senGC maturation. Both Duox2 and its relative Nox1 are ROS-generating NADPH oxidases that are expressed in the intestinal epithelium and have been previously linked to induction by the microbiota (Burgueño et al., 2021; Sommer and Bäckhed, 2015; van der Post et al., 2021). Given that we had observed *Nox1* induction in ConvD adult mice and that both enzymes could theoretically be involved in the senGC activation pathway, we characterized induction of both Duox2 and Nox1 over the course of our different colonization models. Expression of *Duox2* and *Nox1* by qPCR analysis of both ConvD and *B. fragilis* monoassociated tissue demonstrated that both genes were significantly induced in the ConvD, senGC-positive colonization experiment, but were only weakly or not induced in the monoassociated mice (Fig. 7 F). Conversely, analysis of ConvR (P3–P33) and GF (P9–P33) mRNA sequencing data demonstrated microbiota-dependent induction of *Duox2* but not *Nox1* in postnatal tissues (Fig. 7 G).

As functional senGCs have only been observed in the upper crypt epithelium, we next spatially characterized expression of both genes by RNA in situ hybridization in mouse colonic tissue sections (Fig. 7 H). In line with previous observations (van der Post et al., 2021), *Nox1* expression was restricted to the crypt base; however, we observed *Duox2* expression at both the crypt base and upper crypt regions. Immunostaining confirmed Duox2 localization in upper crypt GCs, forming a continuous subcellular pattern likely representing the ER, distinct from glycosylated (UEA1 positive) Muc2 in the Golgi and secretory granules (Fig. 7 I). This pattern was weaker and fragmented in GF mice but restored in ConvD adults. (Fig. 7 I).

To confirm the functional role of *Duox2*, we generated epithelial-specific *Duox2* knockout mice (*Duox2*^ΔIEC^). While *Duox2*^fl/fl^ and *Duox2*^ΔIEC^ littermates exhibited similar baseline mucus secretion, *Duox2*^ΔIEC^ mice lacked the MAMP-induced (LPS and P3CSK4) senGC-dependent response (Fig. 7 J). Histological analysis detected no obvious GC deficiencies that could otherwise explain this loss (Fig. S5 D). Furthermore, blocking *Nox1* with the inhibitor ML171 did not affect MAMP-induced secretion, confirming that *Duox2*, but not *Nox1*, is required for senGC activation. (Fig. 7 K).

These findings demonstrate that microbiota-driven *Duox2* expression is a common feature of postnatal and adult senGC maturation. Given that *Duox2* is now shown as essential for senGC activation, its microbiota-dependent induction likely represents a crucial element in the postnatal development of functional senGCs.

## Discussion

In this investigation, we have defined the postnatal maturation of the colonic IML and senGC protective mechanisms in mixed rodent model systems. We establish that these GC-dependent protective functions develop in sequence in the early pre-weaning environment, with IML barrier function emerging in the immediate postnatal period and IML postsecretory processing dynamics and the senGC response developing subsequently. We further establish that different aspects of this development and GC maturation are regulated either independently or dependently on microbial colonization, with mechanistic roles for MyD88 signaling in modulating postnatal IML proteolytic processing and a causal link between microbiota induction of Duox2 expression and priming of the senGC-dependent secretory response.

As previously established in adult GF mice (Bergstrom et al., 2020; Johansson et al., 2015), we have demonstrated that postnatal development of IML barrier function and fecal encapsulation of the microbiota by Muc2 occurred in a microbiota-dependent fashion that was independent of classical innate immune signaling involving MyD88. Along with previous findings that immune signaling via the inflammasome is not required for baseline IML formation (Bergstrom et al., 2020; Volk et al., 2019), these results further support the concept that microbiota-dependent maturation of this system occurs independently of innate immunity. While our current data does not suggest any candidate regulatory mechanisms, the rapidity of postnatal IML maturation over the first three postnatal days implies that this system develops in response to a well-conserved and fast-acting signal(s), which likely rules out microbial metabolites (e.g., fermentation products) that would take time to build up in the intestinal environment. A possibility that we have not addressed in this investigation is that the colonization status of the mother, rather than incoming microbial colonizers themselves, regulates IML maturation. Prior analysis of small intestinal tissues from GF pups littered by gestationally colonized GF mothers indicates maternal regulation of mucus modulatory ion channel and mucus component genes, including Clca1 and Zg16 (Gomez de Agüero et al., 2016). Examination of colonic IML development in a similar system is a promising future avenue of research.

The postnatal dynamics of IML modulatory factors illustrates a highly mixed landscape of microbiota-dependent and -independent regulation. Critical shapers of Muc2 polymeric network integrity (Tgm3) (Sharpen et al., 2022) and metalloprotease-dependent mucus expansion (Clca1) (Nyström et al., 2018), while strongly age regulated at both the mRNA and protein abundance levels, were not regulated by colonization status in the pre-weaning environment. However, our data does point to the existence of cysteine protease–mediated suppression of mucus expansion in the immediate postnatal period from P1–10, regulated by microbiota colonization via MyD88 signaling. This indicates that the reduced mucus growth observed in this period is partly the result of active suppression by the colonic tissue and correlates with increased mucus thickness observed in vivo. This association makes it tempting to speculate that proteolytic suppression of mucus expansion may reflect a mechanism to slow down mucus turnover during the period that the IML barrier is being established; however, this phenomenon requires further investigation. We furthermore identified microbiota-dependent modulation (both induction and repression) of multiple genes involved in the Muc2 O-glycosylation machinery and GC-fate-targeted transcription factors that were especially regulated in the immediate pre- and postweaning time period. These findings point to a dynamic and coordinated process of postnatal GC and IML maturation that likely reflects the physiological needs of mobilizing intestinal content during the initial switch from in utero to enteral nutrition and subsequently adapting to the significantly increased microbial load and diversity associated with the weaning transition (Bäckhed et al., 2015).

Development of the bacterial MAMP-induced senGC mucus secretory response was observed over the first 1–2 postnatal weeks. Intriguingly, both early neonatal and adult GF colonic tissue responded to MAMPs in a senGC-independent fashion that maintained functional reliance on intact MyD88 signaling but was independent of the active endocytosis and Nlrp6 inflammasome activation required for senGC activation. Histological analysis indicated that senGC-independent responses were more likely the result of fluid rather than mucus secretion, and we speculate that this may function as a rapid response mechanism to flush colonizing bacteria out of developing epithelial crypt structures in the early colon. Intriguingly, the timing of senGC functional maturation was coincidental with postnatal changes in IML properties and increased abundance of key mucus regulatory factors (e.g., Tgm3) that we had previously observed. Our prior work has linked senGCs to a subpopulation of colonic GCs referred to as noncanonical GCs, due to their expression of genes that are normally enriched in nonsecretory epithelial cells. Examination of mucus proteins with increased abundance over the senGC development window identified a distinct noncanonical GC signature in the mucus proteome, including proteins with known (Tgm3 and Lgals3) or putative (Mep1a, Ctsb, Ctsd, and Ctss) roles in regulating mucus protective functions (Bülck et al., 2023; Sharpen et al., 2022). Consequently, our data indicate that senGCs and other noncanonical GCs develop in the pre-weaning environment and may have a direct impact on IML maturation via the production and secretion of mucus regulatory factors that are not expressed by canonical GCs.

Finally, our data demonstrated that postnatal development of functional senGC secretion was driven by microbiota colonization and was likely to be mechanistically driven by induction of Duox2 expression that primes the senGC activation pathway. Previous work has established microbiota regulation of Duox2 expression (Sommer and Bäckhed, 2015) and linked H_2_O_2_ production by Duox2 to activation of TLR signaling (Burgueño et al., 2021), and we now establish a functional role for Duox2 in the senGC activation pathway. Duox2 protein expression in upper crypt GCs was localized to an intracellular structure likely corresponding to the ER or Golgi compartment. Previous work investigating GC uptake of luminal material has found that endocytotic cargo can be trafficked to either of these organelles (Gustafsson et al., 2021), and this Duox2 localization aligns well with our previous work establishing that ROS synthesis functions downstream of endocytosis and TLR signaling in the senGC activation pathway (Birchenough et al., 2016).

The postnatal period represents a crucial window of time where the intestinal mucosa must quickly adapt to both tolerate and defend itself from colonizing microorganisms (Torow and Hornef, 2017). Interruption of this process prevents the tolerogenic imprinting of the mucosal immune system that underlies intestinal homeostasis (Al Nabhani et al., 2019; Knoop et al., 2017), resulting in increased susceptibility to inflammatory disease in later life. GC-intrinsic protective functions are critical legislators of healthy intestinal microbiota–host interactions, and the developmental stages that we have described in this investigation are highly likely to be important aspects of neonatal, infant, and adult health.

## Materials and methods

### Experimental animals

All animals used in this study were housed under specific pathogen–free conditions with ad libitum access to food and water with a 12-h light/dark cycle. All mice were on a C57BL/6N background and bred in-house. Both nonpregnant and timed-mated Wistar rats were purchased from Charles River. GF mice were maintained in sterile flexible film isolators and monitored regularly by aerobic and anaerobic culturing as well as by PCR for bacterial 16S rRNA. The generation of RedMUC2^98tr^ Muc2 reporter mice, *MyD88*^*−/−*^, *Nlrp6*^*−/−*^, and *Duox2* intestinal epithelium conditional knockout mice has been previously described (Adachi et al., 1998; Birchenough et al., 2016; Castrillon-Betancur et al., 2023; Chen et al., 2011). Experimental groups consisted of neonatal, weaned, and adult (12–20-wk-old) mice and rats, as indicated for each experiment. All animals examined were either female only or a balanced mixture of males and females. All experimental groups compared in the study were fed identical rodent chow diets, and all comparisons of WT and null mutant knockout mice used co-housed littermates generated by breeding heterozygous animals. For full conventionalization of GF mice, colonic stool, and cecal content were pooled from three ConvR donor mice and homogenized in PBS supplemented with 1% (wt/vol) cysteine and immediately gavaged (200 μl) into GF recipient mice. Conventionalized mice were subsequently housed under normal ConvR conditions. For monoassociation of GF mice with *B. fragilis*, strain NCTC 9343 was grown at 37°C under anaerobic conditions in BHI broth supplemented with 0.05% (wt/vol) cysteine and 100 nM hemin, and 200 μl of overnight culture was gavaged into GF recipient mice. Monoassociated mice were subsequently housed in sterile isolator cages. All weaned and adult animals were anesthetized using isoflurane and killed by cervical dislocation before collection of samples. All neonatal animals were killed by decapitation. All experimental procedures involving animals were approved by the Swedish Laboratory Animal Ethical Committee in Gothenburg.

### Ex vivo analysis of colonic mucus properties

Colonic mucus thickness, growth rate, and barrier function were determined by using a previously described ex vivo analysis method (Gustafsson et al., 2012). This approach involves mounting live colonic tissues in a horizontal Ussing-like perfusion system that allows direct access to the mucosal side of the tissue via a 1.5–2.5-mm diameter aperture that permits dynamic measurement and imaging of mucus properties in a controlled system. Briefly, fresh tissues were collected by dissection and flushed using cold oxygenated Krebs buffer. Tissues were opened longitudinally, the muscle layer was removed by microdissection, and then mounted mucosa-side up in a custom-made horizontal perfusion chamber with basolateral perfusion of oxygenated Krebs-glucose buffer (10 mM), static apical oxygenated Krebs-mannitol (10 mM), and heated to 37°C. For mucus thickness and growth rate measurements, the mucus surface was visualized using 10-µm black microbeads (Polybead) diluted 1:20 in Krebs-mannitol. Mucus and tissue were observed using a stereomicroscope and the mucus thickness was measured using a 5-µm micropipette linked to a micrometer device (Mitotoyo). Mucus thickness was determined at *t* = 0 min, and mucus growth rate was determined by measuring mucus thickness at *t* = 30 min and *t* = 60 min. In some experiments, tissues were treated with either EDTA (5 mM; Merck), 1× complete EDTA-free protease inhibitor cocktail (Roche), or E64 (20 µM; Merck) inhibitors at *t* = 30 min. For mucus barrier function measurements, tissues were mounted in confocal microscope–adapted imaging chambers without perfusion or heating. Mucus was overlaid with a Krebs buffer solution containing Syto9 tissue dye (12.5 µM; Thermo Fisher Scientific) and 1-µm crimson Fluospheres (1:20; Thermo Fisher Scientific) for 5 min, after which excess dye and beads were removed by washing with Krebs-mannitol. Tissue and Fluospheres were imaged using an LSM700 laser scanning confocal microscope equipped with a 20× water-immersion objective, 488/639-nm lasers, and Zen acquisition software (Carl Zeiss). Fluorescent signals were mapped using Imaris software (Oxford Instruments), and z-axis positions of tissue and Fluospheres were extracted. Mucus barrier function (normalized penetrability) was quantified by analysis of Fluosphere distribution within the mucus layer as previously described (Schroeder et al., 2018; Volk et al., 2019). Frequency distribution curves were generated for each z-stack using Prism 9 software (GraphPad), normalized to maximum frequency values, and then normalized to the position of the mucus surface. Area under the curve data expressed as normalized penetrability were used for quantitative comparison of Fluosphere penetration into the mucus layers of different samples.

### MAMP-induced mucus secretion

The capacity of colonic tissues to secrete mucus in response to bacterial MAMPs was performed using the same ex vivo experimental setup as described above for measuring mucus growth rates with a micropipette-mounted micrometer. All bacterial MAMPs were purchased from InvivoGen and were synthetic or ultrapure grade. Mucus growth was measured at *t* = 0 and *t* = 30 min to establish baseline growth rates. At *t* = 30 min, tissues were treated apically with LPS from *Escherichia coli* O111:B4 (200 µg/ml), LTA from *Staphylococcus aureus* (200 µg/ml), P3CSK4 (50 µg/ml), DNA from *E. coli* (200 µg/ml), flagellin from *Bacillus subtilis* (50 µg/ml), MDP (200 µg/ml), iEDAP (γ-D-glutamyl-meso-diaminopimelic acid; 200 µg/ml), or basolaterally with carbachol (1 mM; Merck). Mucus growth was measured at *t* = 45 min (15 min after treatment) to determine the impact of treatments on growth rate. All MAMPs and carbachol were reconstituted in ddH_2_O, sonicated, and stored at −20°C until use. For experiments using inhibitors to block MAMP-induced mucus secretion, inhibitory compounds were added to apical buffer solutions at *t* = 0 and maintained throughout the experiment. Inhibitors used were Dynasore (100 µM; Merck), Ac-YVAD-cmk Caspase 1/11 inhibitory peptide (100 µM; Merck), and Pepinh-MYD MyD88 inhibitory peptide (100 µM; InvivoGen).

### Mass spectrometry**–**based profiling of the mucus proteome

Samples were collected ex vivo from distal colonic tissues mounted in horizontal perfusion chambers as described above. Mucus was aspirated from the mucosal surface using Maximum Recovery pipette tips (Axygen), mixed with 2x cOmplete protease inhibitor cocktail (Merck), and stored at −80°C until analysis. Sample processing was performed as previously described (Schroeder et al., 2018). Briefly, mucus was reduced overnight in 6 M guanidinium hydrochloride, 0.1 M Tris/HCl (pH 8.5), 5 mM EDTA, and 0.1 M DTT (Merck), followed by filter-aided sample preparation adapted from a previously developed protocol (Wiśniewski et al., 2009) using 10 kDa cut-off filters (Pall Life Sciences). Proteins were alkylated with iodoacetamide (Merck) and sequentially digested on the filter with LysC (Wako) and trypsin (Promega). Peptides were cleaned with StageTip C18 columns prior to MS analysis. 53 NanoLC–MS/MS was performed on an EASY-nLC 1000 system (Thermo Fisher Scientific), connected to a QExactive Hybrid Quadrupole-Orbitrap Mass Spectrometer (Thermo Fisher Scientific) via a nanoelectrospray ion source. Peptides were separated using an in-house packed reverse-phase C18 column with a 60-min 4–32% acetonitrile gradient. Mass spectra were acquired from 320 to 1,600 m/z at a resolution of 70,000, and the 12 peaks with the highest intensity were fragmented to acquire the tandem mass spectrum with a resolution of 35,000 and using automatic dynamic exclusion.

Proteins were identified using MaxQuant (v1.5.7.4) (Cox and Mann, 2008), searching the mouse UniProt protein database supplemented with mouse mucin sequences (https://www.medkem.gu.se/mucinbiology/databases/). Searches used full tryptic specificity, a maximum of 2 missed cleavages, 20 ppm precursor tolerance for the recalibration search followed by 7 ppm for the final search, and 0.5 Da for fragment ions. Modifications were set as carbamidomethylation of cysteine (fixed), methionine oxidation (variable), and protein N-terminal (variable). The false discovery rate (FDR) was set to 1% for both peptide and protein levels, and the minimum peptide length was set to 6 amino acids. Proteins were quantified using label-free quantification using at least two peptides for quantification. Label-free quantification data were analyzed using Perseus (v1.6.2.2) (Tyanova et al., 2016). Proteins were filtered for potential contaminants and detection in at least 50% of samples from one experimental age group. Data were log_10_ transformed, and missing values were imputed from a normal distribution using default settings. Two-sample Welch’s *t* tests with Benjamini–Hochberg FDR were used to identify specific protein abundance differences between experimental groups. Principal component analysis was used to visualize clustering of different sample groups.

For certain analyses, proteins identified by mass spectrometry were defined as “secreted” based on prior knowledge, database annotations, or in silico prediction. Individual protein localization annotations and amino acid sequences were retrieved from UniProt. For in silico prediction of secretion, protein amino acid sequences were analyzed using SecretomeP (v 2.0) (Bendtsen et al., 2004). Predicted classical secretion was defined as the presence of a signal peptide sequence, and predicted nonclassical secretion was defined as the absence of a signal peptide sequence and an neural network (NN) score >0.6.

### Histology

Colonic tissues were fixed in either methanol-Carnoy (Fig. 1, E and L; Fig. 2 C; and Fig. 3 G) or formalin (Fig. 4 I, Fig. 6 K, and Fig. 7 I) solutions for at least 24 h, and tissue was subsequently paraffin embedded and cut into 5-μm thick transverse sections. Tissue sections were deparaffinized by sequential washing in xylene substitute (20 min at 60°C; Merck) and 100% (5 min), 95% (5 min), 70% (5 min), and 30% (5 min) ethanol. For histochemical staining, tissue sections were stained with AB/PAS stains as previously described (Johansson et al., 2008) and imaged using a Nikon Eclipse microscope. Colonic mucus thickness was measured in AB/PAS-stained sections by a blinded investigator recording 10 measurements per tissue section.

For fluorescent staining, antigen retrieval was performed by immersion of sections in 10 mM citrate buffer (95°C, 30 min). Sections were washed in PBS, permeabilized for 5 min using 0.1% vol/vol Triton X-100 (Merck), and blocked using 5% vol/vol FCS. Mature Muc2 and Apo-Muc2 were detected using in-house Muc2C3 (Johansson et al., 2008) and PH497 (Hansson et al., 1994) rabbit polyclonal primary antibodies, respectively. Tgm3 was detected using NBP1–57678 (Novus Biologicals) rabbit polyclonal primary antibody. Duox2 was detected using anti-Duox1/2 I2 (De Deken et al., 2000) rabbit polyclonal primary antibody kindly provided by Professor Francoise Miot (Université libre de Bruxelles, Bruxelles, Belgium). Actin was detected using MAB1501 (Merck) mouse monoclonal primary antibody. Sections were incubated with primary antibodies overnight at 4°C and subsequently washed in PBS and stained with goat anti-rabbit Alexa 488– or Alexa 555–conjugated secondary antibodies or goat anti-mouse Alexa 647–conjugated secondary antibody (Thermo Fisher Scientific) for 2 h at room temperature. Lastly, slides were washed with PBS and counterstained with a Hoechst-34580 DNA dye (5 μg/ml; Merck) in some cases supplemented with combinations of Ulex europaeus agglutinin I (UEA1) Atto 488–conjugated lectin (10 µg/ml; Merck), UEA1 DyLight647–conjugated lectin (10 µg/ml; Vectorlabs), or Wheat germ agglutinin (WGA) Alexa 555–conjugated lectin (10 µg/ml; Thermo Fisher Scientific) for 15 min. Slides were rinsed in dH_2_O, coverslipped using ProLong Gold Antifade mountant (Thermo Fisher Scientific), and imaged using an LSM700 confocal microscope (Zeiss) with a 20× air objective.

For immunohistochemical tissue staining, formalin-fixed tissue sections were stained using the Vectastain Elite ABC Kit mouse IgG kit (#PK-6102; Vector Laboratories) following the manufacturer’s instructions, including antigen retrieval in boiling citrate buffer. For immunostaining of MUC2, we used a 1:1,000 diluted rabbit–anti-Muc2 (#NBP1–31231; Novus Biologicals) primary antibody and a 1:1,000 diluted biotinylated goat–anti-rabbit secondary antibody (#111-065–144; Jackson ImmunoResearch). Slides were visualized using a Zeiss Imager Z1 microscope (Zeiss), and pictures were taken using ZEN pro (Zeiss) software.

For quantitative analysis of epithelial cell numbers in histological tissue sections, Apo-Muc2 staining was used to quantify GCs, and DNA staining was used to quantify total epithelial cells with numbers recorded at all available points around the intestinal tissue section. For quantification of ex vivo tissue responses to P3CSK4, AB/PAS-stained tissue sections were assessed for upper crypt (top 33% of crypt surface-base distance)-filled GC numbers and crypt base cavitation. All histological assessments were carried out by a blinded investigator.

### In situ hybridization

FISH was used to detect bacterial cells and specific mRNA transcripts in histological tissue sections. FISH staining for bacterial 16S rRNA was performed using methanol-Carnoy–fixed tissue sections described above. Sections were deparaffinized by sequential washing in xylene substitute (20 min at 60°C; Merck), 100% ethanol (5 min), and 95% ethanol (5 min). Slides were air-dried and flooded with hybridization buffer (40% vol/vol formamide, 0.1% wt/vol SDS, 0.9 M NaCl, and 20 mM Tris, pH 7.4) supplemented with Alexa 555–labelled universal bacterial FISH probe EUB33840 (1 mM). Slides were incubated at 37°C overnight in a RapidFISH Slide Hybridization Oven (Boekel Scientific), subsequently submerged in wash buffer (0.9 M NaCl and 25 mM Tris, pH 7.4), and incubated for 20 min at 50°C. Lastly, slides were rinsed in double-distilled water and counterstained with Hoechst dye (5 μg/ml; Merck). Stained slides were subsequently imaged using an LSM700 confocal microscope (Zeiss).

FISH staining for mRNA transcripts was performed using the RNAscope technique in combination with formalin-fixed tissue sections. Tissue sections were processed for FISH staining using the RNAscope Multiplex Fluorescent Reagent Kit v2 (Advanced Cell Diagnostics) in combination with probes for either *Duox2* (Mm-Duox2) or *Nox1* (Mm-Nox1) transcripts as per the manufacturer’s instructions. After hybridization, sections were washed three times with 10% vol/vol Tween20-TBS and blocked with 1% wt/vol BSA in Tween20-TBS for 20 min. Sections were counterstained for Epcam by overnight incubation at 4°C with HPA026761 (Merck) rabbit primary antibody. Sections were washed in Tween20-TBS, and primary antibody was detected by incubation with goat anti-rabbit Alexa Fluor 647 secondary antibodies (Thermo Fisher Scientific) for 2 h at room temperature. Stained slides were subsequently imaged using an LSM700 confocal microscope (Zeiss).

### Endocytosis and tissue whole-mount imaging

Colonic tissue was mounted in horizontal perfusion chambers as described above and treated apically with Dextran Alexa 488–conjugated tracer (Thermo Fisher Scientific) for 15 min. Excess tracer was washed away, and the tissue was fixed in formalin for 1 h and then permeabilized with 0.5% (vol/vol) Triton X-100 for 15 min and washed with PBS. Tissue was stained for 1 h with a mixture of Hoechst-34580 DNA dye (10 µg/ml; Merck) and Phalloidin Alexa 647 conjugate (1:400; Thermo Fisher Scientific) to visualize actin. Stained tissue was washed with PBS, transferred to a microscope slide, and coverslipped with Prolong-Gold Antifade mounting medium (Thermo Fisher Scientific). Whole mounts were visualized by generating confocal z-stacks using an LSM 700 microscope (Zeiss) with a 40× oil immersion objective.

Whole mounts of tissue obtained from neonatal and adult RedMUC2^98tr^ mice were prepared by flushing colonic tissue, opening longitudinally and pinning flat to a silicon dissection plate and submerging in formalin for 1 h, followed by staining and confocal imaging as described above. GC numbers per mucosal surface area were quantified by mapping isosurfaces to mCherry fluorescence using Imaris software (Oxford Instruments).

### RNA extraction, qRT-PCR, and sequencing

Distal colonic tissue was fixed for RNA preservation using RNAlater solution (Qiagen). Tissues were lysed in RLT buffer (Qiagen) using an Ultra-Turrax rotor-stator homogenizer (IKA Werke), and RNA was extracted using RNeasy Mini columns (Qiagen) and eluted into RNase-free H_2_O according to the manufacturer’s instructions. The quality of isolated RNA was determined using an Experion Automated Electrophoresis platform (Bio-Rad), and samples were stored at −80°C until further analysis.

Expression of *Nox1* and *Duox2* genes was analyzed by qRT-PCR of cDNA prepared from 600 ng RNA extractions using the High-Capacity cDNA Reverse Transcription Kit (Thermo Fisher Scientific). PCRs (20 μl) were prepared using SsoFast EvaGreen Supermix (Bio-Rad), 450 nM forward and reverse primers, and 10 ng cDNA. PCR cycling conditions were 95°C for 2 min and 40× cycles of 95°C for 5 s and 58°C for 30 s. Reactions were monitored using a CFX96 platform (Bio-Rad) and analyzed using CFX Manager software (v. 3.1; Bio-Rad). Gene expression was quantified using pre-validated PrimePCR primers (Bio-Rad) for *Nox1* (qMmuCED0048182) and *Duox2* (qMmuCID0022771) and the ΔΔCq method with data normalized to the reference genes *Gapdh* (forward primer: 5′-GGA​GAA​ACC​TGC​CAA​GTA​TG-3′; reverse primer: 5′-GGA​GTT​GCT​GTT​GAA​GTC​G-3′) and *Rplpo* (forward primer: 5′-GCG​ACC​TGG​AAG​TCC​AAC​TA-3′; reverse primer: 5′-TCT​CCA​GAG​CTG​GGT​TGT​TT-3′).

RNA sequencing was performed by the Genomics and Bioinformatics Core Facility platforms at Sahlgrenska Academy, University of Gothenburg. The quality of isolated RNA was determined using a Bioanalyzer (Agilent) with minimum acceptable RNA integrity number (RIN) value of 8. cDNA was prepared using the TruSeq Stranded Total RNA Sample Preparation kit with Ribo Zero Gold (Rev. E; Illumina) according to the manufacturer’s protocol and sequenced using a NovaSeq 6000 platform (Illumina). Quality of raw sequencing data was assessed using FastQC (v 0.11.2) (https://www.bioinformatics.babraham.ac.uk/projects/fastqc) and if required, FastQ files were quality filtered using prinseq (v 0.20.3) (Schmieder and Edwards, 2011). Reads were mapped against *Mus musculus* reference genome mm10 using STAR (v 2.5.2b) (Dobin et al., 2013). The alignment quality was assessed using samtools (v 1.6) and qualimap (v 2.2.1) (Li et al., 2009; Okonechnikov et al., 2016). The number of mapped reads toward annotated features in the reference genome was calculated using HTseq (v 0.5.3p3) (Anders et al., 2015).

### RNA sequencing data analysis

Data analyzed in this study included bulk RNA sequencing data generated in the current project supplemented with published bulk RNA sequencing data from sorted colonic GCs and enterocytes and scRNA-seq data of sorted colonic GCs generated as previously described (Nystrom et al., 2021) and deposited under GEO accession number GSE144436.

Statistical analysis and identification of differential gene expression between experimental groups based on bulk RNA sequencing data was performed using DESeq2 (Love et al., 2014) in R. Genes with significant age-correlated monotonic expression patterns were identified by aligning sample age (days) with size factor normalized read counts generated by DESeq2 and calculating Spearman’s rank correlation coefficient. Significant positive and negative age:gene expression correlations were determined after Benjamini–Hochberg FDR correction (Padj < 0.05). For scRNA-seq analysis, demultiplexing, barcoded processing, gene counting, and aggregation were performed using Cell Ranger software (v. 2.1.1) and analyzed using Loupe Browser (v 8.0.0; 10X Genomics). Barcodes were filtered to remove non-GCs based on zero reads of *Ptprc* (immune cells) or *Chgb* (enteroendocrine cells) with *Muc2* reads >3 to avoid enterocyte contamination. Barcodes were further filtered to remove cells with <200 or >5,500 genes detected and mitochondrial gene counts >20%, resulting in 6,123 total cells after filtering. Cells were clustered based on K-means (*K* = 10) and visualized by Uniform Manifold Approximation and Projection (UMAP) embedding. GC cluster identities were defined based on previous analysis and annotation of the same dataset (Nystrom et al., 2021). For mapping of *Rattus norvegicus* genes to *M. musculus* orthologs in scRNA-seq data, *R. norvegicus* gene identifiers were mapped to *M. musculus* identifiers using the g:Orth function of g:Profiler (Kolberg et al., 2023). The mapping pipeline for this analysis is illustrated in Fig. S4 C.

### Microbiota DNA extraction, 16S rRNA gene quantification, and sequencing

Fecal and tissue (intestinal and LN) DNA was extracted using QIAmp PowerFecal Pro kits (Qiagen) with 4x rounds of 4.5 m/s for 40 s bead-beating using a Fast-Prep System (MPBio). Prior to extraction, tissue cells were lysed by brief processing with an Ultra-Turrax rotor-stator homogenizer, followed by pelleting of bacterial cells and tissue debris by centrifugation at 10,000 RCF for 10 min and discarding the supernatant. For absolute quantification of bacterial 16S rRNA gene copy number, DNA extractions were analyzed by qPCR using SsoFast EvaGreen Supermix (Bio-Rad) with 0.3-µm universal 16S primers 926f (5ʹ-AAACTCAAAKGAATTGACGG-3ʹ) and 1062r (5ʹ-CTCACRRCACGAGCTGAC-3ʹ) with 45-ng template DNA. Reactions were performed and monitored using a CFX96 platform (Bio-Rad). Absolute bacterial 16S copy number was quantified using standard curves generated from qPCR of whole 16S gene amplicons purified from *E. coli*, and data were normalized to initial fecal sample mass. For some experiments, the relative proportion of different major bacterial phyla (Bacteroidota, Firmicutes, and Proteobacteria) was determined using taxon-specific primers and PCR conditions as previously described (Bacchetti De Gregoris et al., 2011).

For 16S rRNA gene sequencing, extracted fecal DNA was first subjected to PCR amplification, targeting the V5 and V6 hypervariable regions of the 16S rRNA gene using bacteria-specific primers, with sequences previously described: forward 5′-CCATCTCATCCCTGCGTGTCTCCGACTCAGC-barcode-ATTAGATACCCYGGTAGTCC-3′ and reverse 5′-CCTCTCTATGGGCAGTCGGTGATACGAGCTGACGACARCCATG-3′ (Sundquist et al., 2007). The thermal cycling conditions were set as follows: an initial denaturation at 94°C for 5 min, followed by 35 cycles consisting of denaturation at 94°C for 1 min, annealing at 46°C for 20 s, and elongation at 72°C for 30 s, with final elongation phase at 72°C for 7 min.

PCR amplicons were resolved using a 1% (wt/vol) agarose gel, displaying an anticipated amplicon size of ∼350 base pairs. Following electrophoresis, the amplicons were purified utilizing the QIAQuick Gel Extraction Kit (Qiagen). Quantification of the purified amplicons was performed using the Qubit dsDNA HS Assay Kit on the Qubit 3.0 Fluorometer (Thermo Fisher Scientific). For the preparation of template-positive Ion PGM Template OT2 400 Ion Sphere Particles harboring clonally amplified DNA, we employed the Ion OneTouch Instrument alongside the Ion PGM Template OT2 400 Kit (Thermo Fisher Scientific). Subsequently, sequencing was executed using the Ion PGM Sequencing 400 Kit and Ion 316 Chip V2, within the framework of the Ion PGM System (Thermo Fisher Scientific) (Whiteley et al., 2012).

### 16S rRNA gene sequencing data analysis

FastQ sequencing files produced by the Ion Torrent PGM System were imported into the Quantitative Insights into Microbial Ecology 2 version 2018.8.1 framework (https://qiime2.org/) (Bolyen et al., 2019) and analyzed as previously described (Yilmaz et al., 2019). Within this pipeline, amplicon sequence variants were defined at a 97% sequence identity threshold using the standard settings of Quantitative Insights into Microbial Ecology 2. Taxonomic classification of these amplicon sequence variants was performed utilizing the q2-feature-classifier plugin paired with a Naïve Bayes classifier. The taxonomic classifications were assigned based on their sequence similarity in the SILVA database (https://www.arb-silva.de/).

The features table and mapping file were used to generate a phyloseq object in the R package phyloseq (v 3.4) (McMurdie and Holmes, 2013). The assessment of alpha diversity (Simpson and Shannon indices) and beta diversity (Bray–Curtis genus-level community dissimilarities on principal coordinates analysis) was conducted. Statistical analyses of the clustering patterns were performed using the Mann–Whitney U test for alpha diversity measures and the Adonis test (a type of PERMANOVA) for beta diversity to ascertain the robustness and statistical significance of the group distinctions using identical distance metrics within phyloseq in R. The LEfSe method was used to compare significant differences in taxa between groups (Segata et al., 2011).

### DSS-induced colitis

Colitis was induced in mice by provision of 3% (wt/vol) DSS (TbD Labs) ad libitum in drinking water for up to 7 days. At sacrifice, colonic and lymphatic (CLNs and spleen) tissues were collected and weighed, and colon length was recorded. The most distal 0.5 cm of colonic tissue was separated and snap frozen for 16S rRNA gene quantification (see above). The remaining colon was flushed, opened longitudinally, and rolled into Swiss rolls before fixation in formalin. Fixed Swiss rolls were paraffin embedded, sectioned, and AB/PAS stained as described above. Histopathological assessment of mid-distal colon tissue Swiss roll tissue sections was performed based on an adaptation of a previously defined scoring system assessing inflammatory infiltrate, GC loss, crypt hyperplasia, muscle thickening, submucosal inflammation, abscess formation, and ulceration (Wirtz et al., 2017). Ulcer coverage was determined by measuring the total length of ulcerated mucosal tissue and dividing it by the total length of colonic epithelium in each Swiss roll tissue section. Histology scores were generated by averaging independent scores from two blinded assessors and were summed to generate combined histology scores.

### Online supplemental material

Fig. S1 shows the additional characterization of changes in postnatal mucus barrier properties and proteomic composition. Fig. S2 shows the enlargements and fluorescent signal channel splitting of images shown in Fig. 2 C. Fig. S3 shows the additional characterization of microbiota-induced alterations in GC-enriched gene expression and postnatal changes in GC numbers. Fig. S4 shows the quantitative analysis of images shown in Fig. 4 D and illustrates the analysis pipeline used to generate the data in Fig. 4 G. Fig. S5 shows the sensitivity of senGCs to DSS treatment and tissue histology of Duox2^fl/fl^ and Duox2^ΔIEC^ mice.

## Data Availability

Mass spectrometry proteomics data (related to Fig. 1, G and H; and Fig. S1, C and D) have been deposited to the ProteomeXchange Consortium via the PRIDE partner repository with the dataset identifier PXD052791. Microbiota 16S rDNA sequencing results (related to Fig. 5, B, C, and H–L) have been deposited to the ZENODO repository with the DOI identifier https://doi.org/10.5281/zenodo.13861024. Bulk mRNA sequencing data (related to Fig. 2 D; Fig. 3, C–F; Fig. 7, C–E and G; Fig. S3, A and B; and Fig. 5 C) are deposited in the Sequence Read Archive under accession number PRJNA1235669. Any additional information required to reanalyze the data reported in this paper is available from the lead contact upon request.
