# Correction: Neonatal microbiota colonization primes maturation of goblet cell–mediated protection in the pre-weaning colon

**DOI:** 10.1084/jem.2024159111202025c

**Published:** 2025-11-26

**Authors:** Åsa Johansson, Mahadevan Venkita Subramani, Bahtiyar Yilmaz, Elisabeth E.L. Nyström, Elena Layunta, Liisa Arike, Felix Sommer, Philip Rosenstiel, Lars Vereecke, Louise Mannerås-Holm, Andy Wullaert, Thaher Pelaseyed, Malin E.V. Johansson, George M.H. Birchenough

Vol. 222, No. 8 | https://doi.org/10.1084/jem.20241591 | May 5, 2025

The authors regret that, in Fig. 5, the PCA plot comparison in panel B was originally labeled incorrectly. The plot is between P5 and P15 groups, not between P5 and P10. The original and corrected figures are shown here. This error does not affect the conclusions of the study, and the figure legend remains unchanged. The error appears in print and in PDFs downloaded before November 20, 2025.

**Figure:**
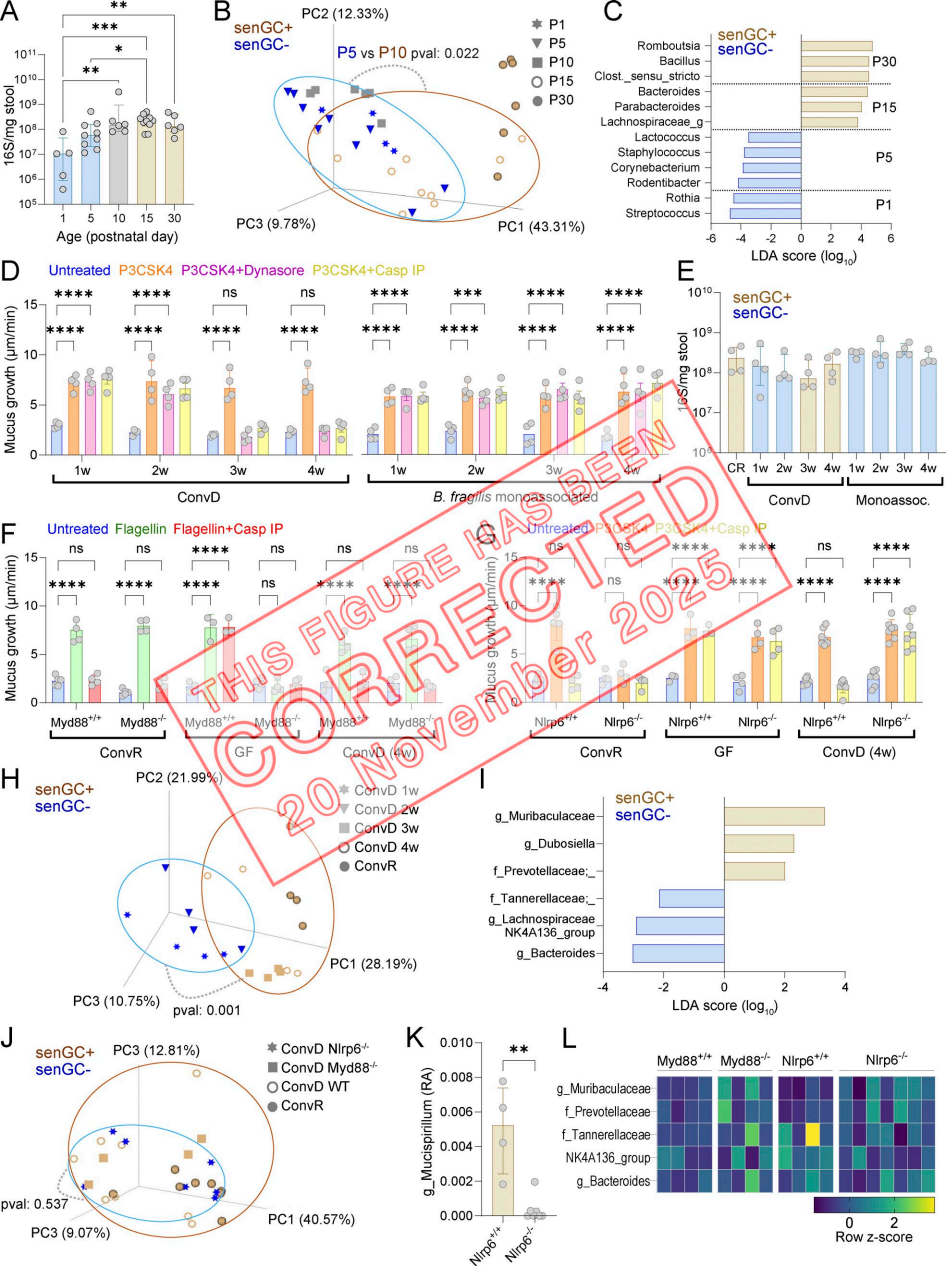


**Figure 5.**
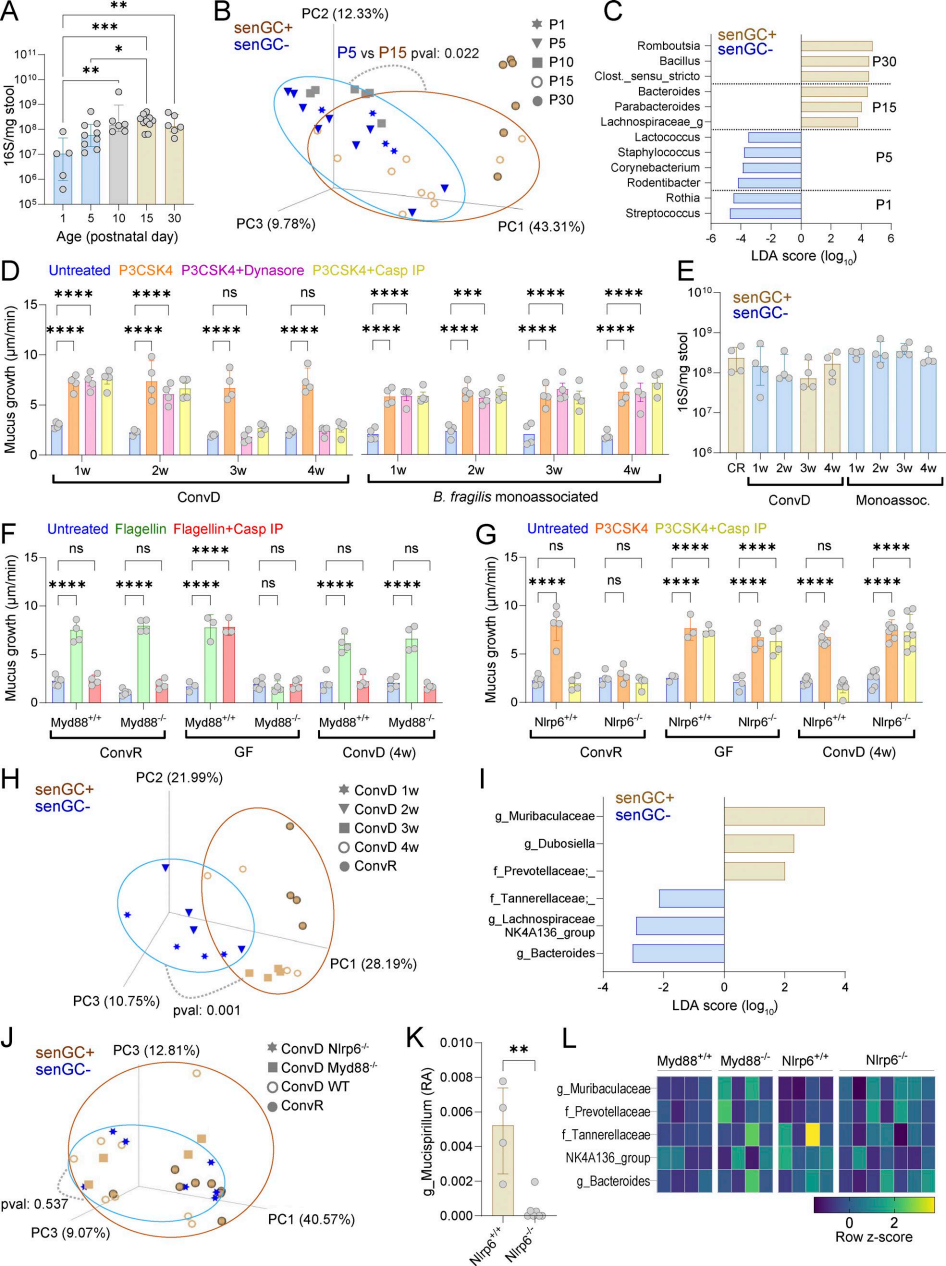
**Microbiota-dependent induction of senGC function. (A)** Total bacterial load in the colon of rats at different postnatal days quantified by 16S qPCR of stool DNA. **(B)** Principal coordinate analysis of postnatal rat microbiota beta diversity (unweighted UNIFRAC) based on metataxonomic 16S sequencing of DNA from stool samples. **(C)** Linear discriminant analysis (LDA) size effect analysis of bacterial taxa significantly enriched in stool from rats at different ages. Taxa enrichment in specific age groups is indicated. **(D)** Ex vivo mucus growth in adult conventionalized (ConvD) and *B. fragilis* monoassociated mouse colon stimulated with P3CSK4 in the presence or absence of senGC activation inhibitors targeting endocytosis (Dynasore) or inflammasome activation (Casp IP). **(E)** Total bacterial load in colon of conventionally raised (CR), ConvD, and monoassociated mice quantified by 16S qPCR of stool DNA. **(F)** Ex vivo mucus growth in adult *MyD88*^+/+^ and *MyD88*^−/−^ ConvR, GF, and 4-wk (w) ConvD mouse colon stimulated with flagellin in the presence or absence of a senGC activation inhibitor targeting inflammasome activation (Casp IP). **(G)** Ex vivo mucus growth in adult *Nlrp6*^+/+^ and *Nlrp6*^−/−^ ConvR, GF, and 4-wk ConvD mouse colon stimulated with P3CKS4 in the presence or absence of a senGC activation inhibitor targeting inflammasome activation (Casp IP). **(H)** Principal coordinate analysis of microbiota beta diversity (Bray–Curtis dissimilarity) based on metataxonomic 16S sequencing of DNA from ConvR and ConvD stool samples. **(I)** Linear discriminant size effect analysis of bacterial taxa significantly enriched in stool from mice with the senGC− or senGC+ phenotype. **(J)** Principal coordinate analysis of microbiota beta diversity (Bray–Curtis dissimilarity) based on metataxonomic 16S sequencing of DNA from ConvR WT, ConvD WT, and ConvD *MyD88*^−/−^ and *Nlrp6*^−/−^ stool samples. **(K)** Relative abundance (RA) of the genus *Mucispirillum* in ConvD *Nlrp6*^+/+^ and *Nlrp6*^−/−^ mice determined by metataxonomic 16S sequencing of stool DNA. **(L)** Standardized abundance (z-score) of bacterial taxa identified in F in 16S sequencing data from ConvD WT and ConvD *MyD88*^−/−^ and *Nlrp6*^−/−^ stool samples. Data represent *n* = 4–9 animals per group, as indicated. All data are pooled from at least two independent experiments or litters. Where relevant (A–C, E, and H–K) experimental groups are color coded by the absence (senGC^−^; blue) or presence (senGC^+^; brown) of the senGC-dependent secretory response. All error-bar graphs show median and interquartile range. Statistical comparisons between groups by two-way ANOVA and Fisher’s LSD (D, F, and G), Kruskal–Wallis and Dunn’s multiple comparison (A and E), PERMANOVA (B, H, and J), or Mann–Whitney test (K); P < 0.05 (*), <0.01 (**), <0.001 (***), <0.0001 (****).

